# Epigenetic Consequences of In Utero PFAS Exposure: Implications for Development and Long-Term Health

**DOI:** 10.3390/ijerph22060917

**Published:** 2025-06-10

**Authors:** Abubakar Abdulkadir, Shila Kandel, Niya Lewis, Oswald D’Auvergne, Raphyel Rosby, Ekhtear Hossain

**Affiliations:** Department of Biological Sciences and Chemistry, Southern University and A&M College, Baton Rouge, LA 70813, USA; abubakar_abdulkad_00@subr.edu (A.A.); shila.kandel@sus.edu (S.K.); niya.lewis@sus.edu (N.L.); oswald_dauvergne@subr.edu (O.D.); raphyel_rosby@subr.edu (R.R.)

**Keywords:** PFAS, in utero, epigenetics, methylation, histone modifications, ncRNAs, placenta, endocrine disruption, developmental effects

## Abstract

In utero exposure to per- and polyfluoroalkyl substances (PFAS) presents significant health concerns, primarily through their role in inducing epigenetic modifications that have lasting consequences. This review aims to elucidate the impact of prenatal PFAS exposure on epigenetic mechanisms, including DNA methylation, histone modification, and non-coding RNA regulation, focusing on developmental and long-term health outcomes. The review synthesizes findings from various studies that link PFAS exposure to alterations in DNA methylation in fetal tissues, such as changes in the methylation of genes like *IGF2* and *MEST*, which are linked to disruptions in growth, neurodevelopment, immune function, and metabolic regulation, potentially increasing the risk of diseases such as diabetes and obesity. We also highlight the compound-specific effects of different PFAS, such as PFOS and PFOA, each showing unique impacts on epigenetic profiles, suggesting varied health risks. Special attention is given to hormonal disruption, oxidative stress, and changes in histone-modifying enzymes such as histone acetyltransferases (HATs) and deacetylases (HDACs), which are pathways through which PFAS influence fetal development. Additionally, we discuss PFAS-induced epigenetic changes in placental tissues, which can alter fetal nutrient supply and hormone regulation. Despite accumulating evidence, significant knowledge gaps remain, particularly regarding the persistence of these changes across the lifespan and potential sex-specific susceptibilities. We explore how advancements in epigenome-wide association studies could bridge these gaps, providing a robust framework for linking prenatal environmental exposures to lifetime health outcomes. Future research directions and regulatory strategies are also discussed, emphasizing the need for intervention to protect vulnerable populations from these environmental pollutants.

## 1. Introduction

Per- and polyfluoroalkyl substances (PFAS) encompass a diverse class of synthetic chemicals that have been extensively utilized since the 1940s in industrial applications and consumer products, ranging from non-stick cookware to firefighting foams [1]. Their resilience stems from robust chemical bonds that confer exceptional chemical and thermal stability, ultimately resulting in pronounced environmental persistence and bioaccumulation [2]. PFAS have thus earned the moniker “forever chemicals,” as their exceedingly long half-lives ensure they remain in ecosystems and human tissues for prolonged periods [3,4,5]. These characteristics have sparked global public health concerns, especially for vulnerable populations such as pregnant individuals. Indeed, recent biomonitoring efforts indicate that a majority of pregnant women in developed countries show detectable PFAS levels in serum or cord blood, with some studies reporting detection rates above 90% [6,7,8,9]. Such widespread detection underscores the urgency of understanding how prenatal exposures may perturb critical developmental processes.

Recent regulatory measures and manufacturing modifications have shifted the usage patterns of some PFAS, resulting in decreased levels of “legacy” compounds such as perfluorooctane sulfonate (PFOS) and perfluorooctanoic acid (PFOA) in certain regions [10]. Nevertheless, a growing roster of new PFAS analogs with varying chain lengths and functional groups continues to be introduced, perpetuating the need for ongoing surveillance. The complex landscape of emerging PFAS, coupled with inconsistencies in analytical detection methods and exposure assessment intervals, can yield divergent findings across epidemiological studies. These discrepancies highlight both the technical and interpretative challenges inherent to PFAS research and underscore the importance of standardizing exposure protocols to enable valid cross-study comparisons.

In parallel with evolving exposure profiles, mounting interest has arisen regarding the epigenetic consequences of in utero PFAS exposure. Epigenetic mechanisms encompassing DNA methylation, histone modifications, and non-coding RNAs regulate gene expression during embryogenesis and fetal development, orchestrating essential processes in organogenesis while rendering the developing epigenome particularly vulnerable to environmental perturbations [11,12,13,14,15]. PFAS exposure is especially concerning, as the fetal epigenome is exceptionally malleable during critical developmental windows when disruptions to processes such as imprinting establishment and chromatin remodeling can have enduring consequences [16,17].

A growing body of evidence suggests that PFAS exposures during pregnancy can alter various layers of epigenetic regulation, sometimes in a coordinated manner [16,18]. For instance, DNA methylation changes at imprinted loci, including Insulin-like growth factor 2 (IGF2) and mesoderm-specific transcript (MEST), have been associated with metabolic phenotypes and growth patterns later in life [16,19,20]. Parallel alterations in histone acetylation and methylation patterns in PFAS-exposed tissues [21] indicate that PFAS could modify the enzymatic machinery involved in chromatin remodeling. Non-coding RNAs, particularly microRNAs (miRNAs) and long non-coding RNAs (lncRNAs), have further expanded our understanding of how PFAS may influence gene expression networks.

Despite the accumulation of supportive data, significant uncertainties remain regarding the precise causal pathways linking prenatal PFAS exposures to specific health outcomes. Many studies are observational in nature and may be confounded by demographic or socioeconomic differences, concomitant exposures to other endocrine-disrupting chemicals, and incomplete characterization of PFAS mixtures [22,23]. Equally important, there is a need to clarify whether molecular differences among PFAS of varying chain lengths and functional groups lead to distinct epigenetic footprints. Emerging research also suggests potential sex-specific responses, wherein male and female fetuses exhibit differential epigenetic susceptibilities, adding an additional layer of complexity that warrants systematic investigation [3,16].

In this review, we aim to deliver a nuanced and forward-looking synthesis of how prenatal exposure to PFAS orchestrates epigenetic regulation and shapes health trajectories across the lifespan. By exploring the mechanisms through which PFAS disrupt epigenetic regulation, including DNA methylation, histone modifications, and non-coding RNA networks, this review will evaluate how the diverse PFAS compounds influence these molecular pathways. It will further examine prenatal epigenetic programming during critical developmental windows, emphasizing the susceptibility of placental and embryonic tissues to PFAS-induced alterations and their role in driving sex-specific vulnerabilities and potential transgenerational health risks. The review will assess the spectrum of adverse health outcomes linked to prenatal PFAS exposure, such as preeclampsia, low birth weight, neurodevelopmental disorders, and chronic cardiometabolic diseases, while delineating the epigenetic pathways that mediate these effects. Additionally, it will analyze the long-term consequences of developmental PFAS exposure, including persistent metabolic reprogramming (e.g., obesity, type 2 diabetes), immune dysfunction, and neurodevelopmental impairments, underscoring how early-life epigenetic changes may perpetuate disease risk into adulthood. By integrating mechanistic insights, epidemiological findings, and translational evidence, this review seeks to identify critical gaps in understanding PFAS-epigenome interactions, inform public health policies to mitigate exposure risks, and guide future research toward interventions that address the epigenetic legacy of PFAS across the lifespan.

## 2. Mechanisms of Epigenetic Modification of PFAS-Induced Disruptions

### 2.1. Properties, Structure, and Epigenetic Fundamentals of PFAS

The PFAS are a diverse group of synthetic fluorinated organic chemicals recognized for their exceptional chemical stability. They have been widely employed in industrial and consumer products, including firefighting foams, nonstick cookware, water- and stain-resistant coatings, and food packaging [24,25]. This widespread presence is due to the strong carbon-fluorine (C–F) bond, one of the strongest in organic chemistry, with a bond dissociation energy of approximately 485 kJ/mol [26]. Fluorine’s high electronegativity and tight orbital overlap with carbon’s 2p orbitals yield structures that are both hydrophobic and lipophobic traits that make PFAS ideal for myriad applications but also render them resistant to hydrolysis, photolysis, and biodegradation [25,27]. Furthermore, within the broad PFAS family, partially fluorinated compounds, often referred to as polyfluoroalkyl substances, undergo metabolic or environmental conversion to form perfluoroalkyl acids (PFAAs). This transformation extends the exposure timeline, thereby opening multiple windows, both direct and indirect, during which epigenetic regulatory mechanisms may be perturbed [28]. For instance, while initial exposure to poly-fluoroalkyl substances might not be as prolonged, the cumulative body burden of resultant PFAAs can sustain epigenetic changes during critical developmental periods [25,29,30].

Epigenetic regulation constitutes a complex network of heritable modifications that modulate gene expression via dynamic chemical marks deposited on DNA and histone proteins without altering the underlying nucleotide sequence [31,32]. Among these processes, DNA methylation, the covalent addition of a methyl group to the fifth carbon of cytosine residues within CpG dinucleotides, stands as one of the most extensively characterized mechanisms. By influencing chromatin structure and the accessibility of transcriptional machinery, precise DNA methylation patterns are critical for orchestrating developmental programs and maintaining cellular homeostasis. Disruption of these patterns, particularly during sensitive developmental windows, may lead to aberrant gene expression and predispose individuals to a range of adverse health outcomes. Evidence indicates that prenatal PFAS exposure can perturb not only DNA methylation but also other epigenetic regulatory pathways, including histone modifications and non-coding RNA (ncRNA)-mediated processes [33,34]. These mechanisms are orchestrated by a dynamic interplay of “writers,” “erasers,” and “readers” that collectively establish and interpret the epigenetic landscape [35,36,37]. Given that prenatal development represents a period of heightened sensitivity, PFAS-induced epigenetic disruptions may impart enduring modifications with potential implications for organogenesis, metabolic homeostasis, and long-term disease risk.

### 2.2. PFAS Exposure and DNA Methylation: A Nexus of Environmental and Epigenetic Impact

DNA methylation involves the enzymatic addition of a methyl group to the fifth carbon position of cytosine residues within CpG dinucleotides, yielding 5-methylcytosine (5-mC) [36,38]. Notably, these methylation marks are relatively stable and mitotically heritable, exerting control over gene expression without modifying the underlying DNA sequence [20]. The establishment and maintenance of methylation patterns are orchestrated by DNA methyltransferases (DNMTs), an enzyme responsible for adding methyl groups to DNA: DNMT3A and DNMT3B mediate de novo methylation, while DNMT1 preserves these patterns during DNA replication [39,40]. Although promoter and enhancer methylation typically correlate with transcriptional repression, the dynamic action of ten-eleven translocation (TET) enzymes, which oxidize 5-mC to 5-hydroxymethylcytosine (5-hmC), ensures that methylation is a reversible and finely tuned process [41,42]. It is important to note that while 5-mC is crucial for processes such as X-chromosome inactivation, genomic imprinting, and suppression of transposable elements, its oxidation product, 5-hmC, plays a particularly pivotal role during early development. This includes reprogramming DNA methylation patterns following fertilization and in primordial germ cells, thereby distinctly contributing to cellular differentiation and neural development [43]. In the context of in utero PFAS exposure, these precisely regulated methylation dynamics may be perturbed through direct interference with DNMT and TET activities, alterations in one-carbon metabolism, or the induction of oxidative stress, thereby establishing aberrant methylation profiles during key developmental windows [33,34,44].

Shortly after fertilization, the paternal genome undergoes rapid, active demethylation, while the maternal genome is passively demethylated over several cell divisions [45,46,47]. By the time the embryo implants, a wave of de novo methylation reinstates tissue- and lineage-specific methylation patterns [46]. These marks help control gene activity, genomic imprinting, transposon silencing, and X-chromosome inactivation. In parallel, specialized regions known as differentially methylated regions (DMRs) are maintained in a parent-of-origin manner, ensuring proper imprinting and dosage of critical developmental genes [46,48]. Notably, early embryogenesis is characterized by two distinct waves of epigenetic reprogramming. The first wave, immediately following fertilization, involves the erasure of parental methylation marks and the establishment of a somatic epigenetic profile in the developing embryo. The second wave, occurring in fetal primordial germ cells, involves tightly regulated de novo re-methylation that re-establishes imprinted and germline-specific marks in a sex- and parent-of-origin-dependent manner [49]. These processes are further modulated by histone post-translational modifications and non-coding RNAs, which collaborate with DNA methylation to enforce silencing at imprinted loci and transposable elements [43,50]. The precise regulation of these reprogramming waves is essential for establishing correct genomic imprinting, transposon silencing, and X-chromosome inactivation. The disruption of these events by PFAS exposure could potentially lead to aberrant methylation patterns, thereby compromising the fidelity of developmental gene regulation and predisposing the organism to later-life health complications. This intricate choreography of DNA methylation shapes key developmental decisions and, importantly, creates windows of susceptibility where environmental exposures such as PFAS can alter the epigenetic landscape [20,46].

**Figure 1:**
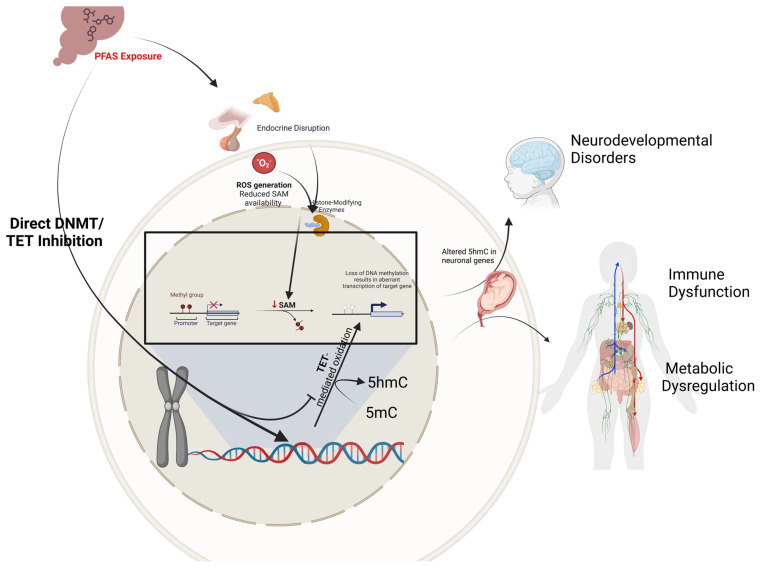
Schematic illustration of PFAS-induced DNA methylation and associated health outcomes. This figure depicts how PFAS interferes with normal DNA methylation processes and leads to downstream pathophysiological effects. At the cellular level, PFAS exposure is shown to induce reactive oxygen species (ROS) generation and endocrine disruption, while directly inhibiting the activity of DNA methyltransferases (DNMTs) and ten-eleven translocation (TET) enzymes. Within the nucleus, the black inset highlights the enzymatic conversion of 5-methylcytosine (5-mC) to 5-hydroxymethylcytosine (5-hmC) by TET. PFAS-mediated inhibition of DNMT/TET can result in aberrant methylation patterns that dysregulate gene expression. These epigenetic perturbations are linked to a variety of adverse health outcomes, including neurodevelopmental disorders, immune dysfunction, and metabolic dysregulation. Arrows and text boxes illustrate the mechanistic flow from PFAS exposure to disrupted epigenetic marks, underscoring critical windows of vulnerability during early development.

PFAS has been shown to alter DNA methylation patterns and exert long-lasting effects on gene function and health outcomes (Figure 1) [20,51]. This effect occurs via multiple mechanisms, one of which involves disrupting DNMT activity. By inhibiting or altering DNMT activity, PFAS can lead to hypomethylation or hypermethylation of specific genomic regions, resulting in dysregulated gene expression [52]. Additionally, the association of PFAS exposure with increased oxidative stress can indirectly affect DNA methylation [53,54]. Oxidative stress can lead to DNA damage and alter the availability of S-adenosylmethionine (SAM), the primary methyl donor in methylation reactions, impacting global methylation levels [55]. Furthermore, as endocrine disruptors, PFAS can interfere with hormonal pathways crucial for regulating genes involved in growth, metabolism, and development [56]. Hormonal imbalances may influence the expression of genes encoding DNMTs and other epigenetic modifiers, leading to aberrant methylation patterns [57,58,59]. During fetal development, precise DNA methylation is essential for organogenesis and neural development [60,61]. Epigenetic modifications affecting genes involved in lipid metabolism, glucose homeostasis, and insulin signaling may contribute to metabolic syndromes. Aberrant methylation of these genes can lead to obesity, type 2 diabetes, and dyslipidemia [61]. Furthermore, changes in DNA methylation can alter the expression of cytokines and other immune-related genes, potentially leading to immunosuppression or autoimmunity [62,63]. This dysregulation increases susceptibility to infections and may reduce the efficacy of immune responses [60].

Several studies have investigated the relationship between PFAS exposure and DNA methylation, providing insights into the epigenetic mechanisms underlying PFAS-associated health effects. One such study by Liu and co-workers (2022) [61] examined how prenatal PFAS exposure affects DNA methylation at birth and early adolescence [61]. Their study identified persistent DNA methylation changes associated with gestational exposure to different PFAS, including 413 CpG sites for perfluoro nonanoic acid (PFNA), 12 for PFOA, eight for perfluorohexane sulfonic acid (PFHxS), and two for PFOS, observed at both birth and 12 years of age. These methylation changes are mapped to genes linked to critical health outcomes, including cancer, cardiovascular health, cognitive function, and kidney function. Different PFAS compounds showed unique methylation patterns with minimal overlap, suggesting distinct biological impacts of each type. For example, PFNA was associated with the greatest number of altered CpG sites. Enrichment analysis revealed that these methylated CpG sites were predominantly involved in pathways related to cell adhesion, immune function, and intracellular signaling, all crucial for proper tissue and immune development [61]. The preserved nature of these epigenetic modifications implies that exposure during gestation could establish lasting DNA methylation patterns that impact gene expression across various biological pathways. This “fetal programming” effect highlights the potential for early-life PFAS exposure to influence disease risk later in life.

Similarly, Petroff and colleagues (2023) [20] explored how PFAS exposure impacts birth outcomes via epigenetic alterations, focusing on 5-mC DNA methylation and 5-hmC hydroxymethylation. The study observed that exposure to PFAS led to widespread changes in both DNA methylation and hydroxymethylation at multiple CpG sites in umbilical cord blood DNA, with the main findings including the prevalence of 5-hmC changes being more compared to 5-mC changes, indicating that PFAS might hinder active regulatory processes during fetal development [20]. Furthermore, decreased 5-hmC levels were found at genes involved in neurodevelopment and metabolic regulation, such as *SHANK2* (linked to synaptic signaling) [64] and *MYH9* (involved in cell motility) [65]. PFAS exposure exhibited sex-specific effects, affecting different CpG sites in male and female infants, which could contribute to varying susceptibility to adverse health outcomes depending on sex. Importantly, changes in 5-mC and 5-hmC mediated the relationship between PFAS exposure and adverse birth outcomes, such as shorter gestational periods and lower birth weight, indicating an increased risk for preterm births. The study suggests that PFAS exposure might suppress TET enzymes, which mediate the conversion of 5-mC to 5-hmC, leading to reduced levels of 5-hmC and impairing regulatory processes during fetal development [66]. As acknowledged by the authors [20,61], and as with other observational birth-cohort studies [34], these associations remain correlational: uncontrolled exposure assessment and potential unmeasured confounders limit causal inference in the absence of complementary mechanistic validation.

Additionally, a study conducted by Everson and co-workers (2025) [17] examined how PFAS exposure in the placenta affects DNA methylation at loci associated with cardiometabolic health. The researchers conducted an epigenome-wide association study on placental tissue, focusing on five PFAS compounds [17]. They found that PFHxS had the most significant effect, linked to 11 differentially methylated loci. Other PFAS were also linked to methylation changes in genes involved in growth processes, cardiometabolic health, and neurodevelopment. Alterations in methylation were enriched in pathways associated with the biosynthesis of branched-chain amino acids, crucial for energy homeostasis, and linked to cardiometabolic diseases such as obesity and insulin resistance. Sex-specific analyses suggested that females might be more vulnerable to PFAS-induced methylation changes, indicating potential sex-specific epigenetic vulnerabilities. These methylation changes directly affect genes involved in cardiometabolic regulation and growth processes, which can have downstream health implications. The findings suggest a potential mechanism through which PFAS exposure during pregnancy could increase the risk of metabolic diseases and developmental disorders in offspring. These studies highlight the significant impact of PFAS exposure on DNA methylation epigenetic mechanisms, leading to alterations in gene expression regulation that can have profound effects on health outcomes. A summary of key human cohort studies investigating prenatal PFAS exposure and DNA methylation alterations is presented in Table 1. However, as a single-cohort observational EWAS, these findings remain correlative despite pooling tissue from six maternal and fetal sites and adjusting for estimated cell-type proportions via the planet deconvolution algorithm, residual confounding from placental heterogeneity may persist. Furthermore, while the authors performed rigorous sensitivity analyses (alternative imputation methods, mixture modeling, eQTM), the absence of orthogonal mechanistic validation (e.g., functional assays or in vivo models) precludes causal inference. The modest overall and subgroup sample sizes also limit statistical power, underscoring the need for replication in larger, more diverse cohorts.

### 2.3. PFAS Exposure and Histone Modifications: Epigenetic Regulators of Developmental Toxicity

In addition to alterations in DNA methylation, PFAS exposure has been shown to target another fundamental epigenetic mechanism: histone modifications. The Eukaryotic DNA is intricately packaged around histone proteins (H2A, H2B, H3, and H4), and the dynamic addition or removal of chemical groups such as acetyl, methyl, phosphate, ubiquitin, or small ubiquitin-related modifier (SUMO) on histone tails plays a critical role in regulating chromatin structure and gene expression [35,50]. For instance, histone acetylation generally promotes an open chromatin conformation conducive to active transcription, whereas specific histone methylation marks are associated with gene repression [50]. Additionally, the deposition and exchange of histone variants, such as H2A.Z or H3.3, also influence nucleosome stability and transcriptional activity processes that are prone to disruption when PFAS alters the normal deposition, recognition, or turnover of histone variants [74]. Emerging evidence indicates that PFAS compounds can disrupt this balance by directly inhibiting histone-modifying enzymes or indirectly altering their function via endocrine disruption and oxidative stress [33,44,75]. Critically, PFAS-induced signaling aberrations may also impinge upon ATP-dependent chromatin remodelers, such as the SWI/SNF and CHD complexes, thereby modulating the exposure of regulatory DNA elements [31]. This multifaceted interference with chromatin dynamics contributes to the epigenetic deregulation observed during critical developmental windows.

**Figure 2:**
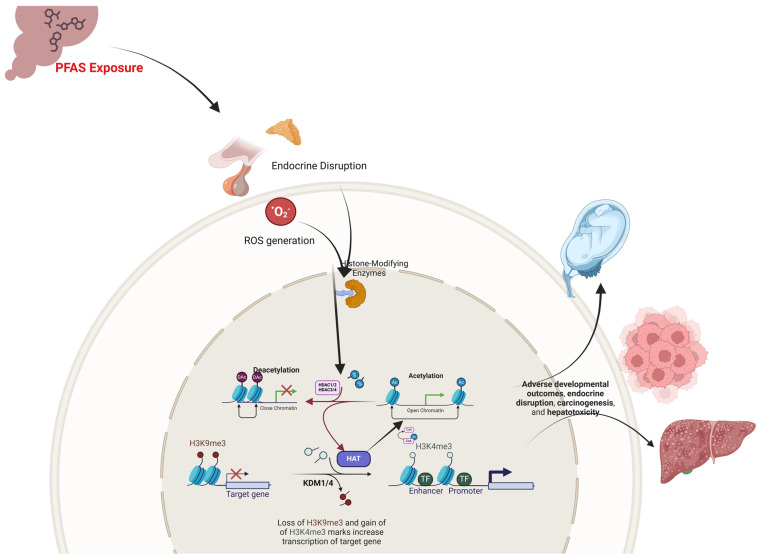
Proposed mechanistic model of PFAS-induced epigenetic disruption. The schematic illustrates how PFAS, such as PFOS and PFOA, can alter histone modifications and downstream developmental processes. Top left: PFAS exposure initiates endocrine disruption and reactive oxygen species (ROS) generation. Center (cell nucleus inset): Within the nucleus, PFAS targets histone-modifying enzymes specifically histone acetyltransferases (HATs) and histone deacetylases (HDAC1/2, HDAC3/4), leading to aberrant acetylation states (increased or decreased histone acetylation). PFAS-induced oxidative stress and metabolic disturbances further deplete or dysregulate cofactors (e.g., acetyl-CoA, S-adenosylmethionine), thereby influencing both acetylation and methylation marks. The figure depicts a loss of H4K20me3 (a repressive methyl mark) and a gain of H4K16ac (an activating acetyl mark) as representative examples of PFAS-driven chromatin remodeling. These modifications collectively promote open chromatin conformations at key regulatory loci, resulting in dysregulated transcription. Surrounding icons highlight the downstream impacts on critical biological systems: (i) developmental processes in the embryo, (ii) endocrine function in hormone-producing tissues, (iii) metabolic homeostasis in the liver, and (iv) potential tumorigenic changes in proliferating cells. Together, these observations underscore how PFAS exposures, through direct enzyme inhibition and indirect metabolic/oxidative pathways, compromise chromatin architecture during vulnerable developmental windows, with long-term implications for disease etiology.

Research has highlighted the significant role of PFOS and PFOA in disrupting histone modifications, a pivotal component of chromatin regulation that governs gene expression during development (Figure 2). These compounds exert their effects by perturbing the activity of histone-modifying enzymes, including histone acetyltransferases (HATs) and histone deacetylases (HDACs). PFAS-induced oxidative stress and metabolic disturbances further contribute to this dysregulation by altering the intracellular pools of key cofactors (e.g., acetyl-CoA and S-adenosylmethionine) necessary for the proper execution of post-translational modifications on histone tails. In essence, the convergence of direct enzyme interactions, reactive oxygen species (ROS)-mediated modifications, and metabolic shifts establishes a common mechanistic framework by which PFAS compromise chromatin architecture, thereby perturbing gene transcription during critical developmental windows [18,21,50].

Building on these mechanistic insights, several studies have detailed how alterations in HAT and HDAC activities manifest in adverse developmental outcomes. PFOS, characterized by its sulfonate moiety, has been shown to selectively impair HDAC1 and HDAC2 function, leading to increased histone acetylation and subsequent aberrant activation of genes critical for cell cycle progression and tissue patterning [44,76]. In contrast, with its carboxylate group, PFOA exerts a broader inhibitory effect on multiple HDAC isoforms, including HDAC3 and HDAC4, thereby influencing a wider array of embryonic genes that regulate tissue specification and metabolic homeostasis [77]. These disruptions in the balance of histone acetylation and deacetylation are particularly detrimental during the transition from maternal to zygotic gene control, where precise histone modifications such as H3K4me3 and H3K9me3 are essential for orchestrating gene activation and repression during early embryogenesis [78,79].

Beyond the immediate developmental milieu, PFAS-induced histone modifications have been implicated in long-term endocrine and metabolic disturbances. For example, Alam and colleagues (2021) [80] demonstrated that low-level, chronic PFOS exposure upregulated HAT activity, specifically increasing H3K9 and H3K18 acetylation at promoters of key steroidogenic genes [80]. This modulation correlated with enhanced testosterone and progesterone biosynthesis, suggesting a direct link between PFAS-induced histone acetylation and endocrine disruption. Notably, because this work was performed in a controlled rodent model that may not fully recapitulate human gestational exposure, and no HAT-inhibition experiments were conducted, it remains unclear whether the acetylation changes drive hormone production or simply accompany altered transcriptional states. Such alterations in the epigenetic landscape not only compromise organogenesis but also predispose the developing organism to metabolic and neurodevelopmental disorders later in life [32,34].

PFAS-associated histone modifications have also been associated with carcinogenic processes, particularly in breast epithelial cells. In human breast epithelial cells (MCF-10A), PFOS exposure markedly reduced H3K9 acetylation, thereby compacting chromatin and potentially silencing tumor suppressor genes [81]. Concurrently, PFOA exposure was linked to reduced H3K9 dimethylation and H3K4 trimethylation across cell generations, alterations that may lift repressive marks and facilitate oncogenic transformation. Complementing these findings, both PFOS and PFOA were observed to elevate global DNA methylation levels, an epigenetic alteration that could further silence critical regulatory genes involved in cell cycle control and apoptosis. These findings underscore a potential mechanism by which transient PFAS exposures engender heritable epigenetic changes, ultimately increasing cancer risk [81]. However, because Pierozan et al. (2020) [81] conducted these experiments in an immortalized breast epithelial cell line, extrapolating the results to in vivo human cancer risk must be done cautiously. The artificial high-dose exposure conditions and lack of systemic context mean that the epigenetic alterations observed, while mechanistically intriguing, remain correlative; without in vivo validation (for instance, in animal models or human tissues), it is unproven that these histone changes actually drive carcinogenesis.

In hepatotoxicity, maternal PFOA exposure has been implicated in epigenetically mediated liver dysfunction in offspring. Li and co-workers (2019) [82] reported that reduced HAT activity coupled with heightened HDAC activity in the liver resulted in decreased histone H3/H4 acetylation [82]. This chromatin compaction was associated with diminished expression of peroxisome proliferator-activated receptor alpha (PPAR-α), a key regulator of lipid metabolism, and a paradoxical upregulation of specific downstream targets that may exacerbate oxidative stress. Histopathological analyses further confirmed the presence of hepatocyte swelling and vacuolar degeneration, emphasizing the multifaceted impact of PFAS on liver physiology via epigenetic mechanisms. Nevertheless, Li et al. relied exclusively on transiently transfected HEK-293 cells exposed to micromolar PFOA, evaluated only a limited PFAS congeners panel, and offered no primary-hepatocyte or in vivo validation; these in vitro constraints temper confidence in the findings’ applicability to human gestational exposures.

The collective evidence highlights that while PFOS and PFOA share common mechanistic pathways in disrupting histone modifications, the nuances of their effects vary according to their chemical structure and subsequent interactions with specific epigenetic regulators. Notably, recent studies comparing PFAS with their replacement analogs, such as hexafluoropropylene oxide trimer acid (HFPO-TA), reveal that alternative compounds may induce even more pronounced epigenetic changes, challenging the notion of their relative safety [83].

### 2.4. Non-Coding RNA Networks in PFAS-Mediated Epigenetic Perturbations

ncRNAs, including miRNAs and lncRNAs, are fundamental in regulating gene expression and chromatin organization, and their dysregulation under PFOS exposure provides critical insights into the epigenetic toxicity of PFAS [84,85,86,87]. During embryonic development, spatiotemporal expression of specific ncRNAs helps shape critical gene regulatory networks. For example, lncRNAs such as XIST (X-inactive specific transcript) are essential for X-chromosome inactivation, while a broad repertoire of miRNAs orchestrates lineage commitment by regulating both transcription factors and downstream effector genes [88]. Environmental stressors that alter ncRNA profiles may disrupt these highly coordinated pathways. Growing evidence suggests that PFAS exposure can shift miRNA expression levels, potentially influencing placental development and embryonic growth [17].

Li and colleagues (2022) [87] highlighted a crucial role for lncRNA MEG3 (maternally expressed gene 3) in PFOS-induced placental dysfunction, wherein MEG3 expression is significantly downregulated in both PFOS-exposed mice and human trophoblast cells, correlating with increased DNA methylation at its promoter [87]. This suppression of MEG3 transcription underscores DNA methylation as a key contributor to placental toxicity, given MEG3’s typical functions in tumor suppression and placental health. Intriguingly, miR-770, which is derived from the intronic region of MEG3, also shows reduced expression under PFOS exposure, suggesting that MEG3 positively regulates miR-770, with both molecules mitigating PFOS-induced placental cell growth inhibition [87]. Mechanistically, the MEG3/miR-770 axis targets PTX3 (Pentraxin 3), an inflammatory and developmental regulator that is overexpressed in PFOS exposure, further contributing to placental cell growth inhibition. However, suppressing PTX3 through miR-770 overexpression or PTX3-specific siRNA alleviates the toxic effect, pinpointing PTX3 as a critical downstream mediator of PFOS-induced placental dysfunction. These findings delineate a lncRNA–miRNA–mRNA regulatory network (MEG3/miR-770/PTX3) whose disruption by PFOS leads to adverse outcomes in the placenta and offer a potential avenue for therapeutic interventions, such as demethylation of MEG3 or modulation of the miR-770/PTX3 pathway [87]. However, because these findings derive from rodent in vivo and trophoblast in vitro models, their relevance to human gestation remains to be established; species-specific differences and the complexity of maternal–fetal interactions may modulate this epigenetic circuit.

Beyond this placenta-specific MEG3/miR-770 mechanism, broader epigenomic surveys in PFOS-exposed MCF-10A cells revealed 494 differentially methylated lncRNAs, comprising 20.5% of all affected genes, with a predominance of hypermethylation events in promoters and CpG islands [89]. LncRNAs such as GACAT3, DELEC1, CASC2, and LCIIAR exhibited altered methylation status under PFOS exposure, suggesting potential disruption of tumor-suppressive functions or promotion of malignant phenotypes [89,90]. For instance, GACAT3, generally associated with elevated expression in breast cancer tissues, was hypermethylated despite its usual upregulation in malignancies, whereas DELEC1, a known tumor suppressor, exhibited hypermethylation correlating with reduced gene activity [89]. Similarly, CASC2 and LCIIAR showed hypermethylation events that, in various cancers, are linked to aberrant cell proliferation and tumor progression [91,92]. These data illustrate the complexity of PFOS-induced epigenetic reprogramming, where promoter hypermethylation can sometimes yield context-dependent changes rather than straightforward gene silencing.

Alongside lncRNAs, miRNAs are also subject to PFAS-mediated epigenetic perturbations, either directly or through DNA methylation changes at miRNA promoters. PFAS exposure has been shown to repress miRNAs such as miR-101-3p, miR-144-3p, and miR-19a-3p, all of which play significant roles in carcinogenesis and cardiovascular function [93]. Comparable effects have been observed with structurally related toxicants like dioxins, which alter miR-101a and miR-122 levels important for hepatic function and cancer pathways [94]. In cardiovascular contexts, the repression of miR-19a-3p can exacerbate inflammatory responses and disrupt normal development, thereby intensifying susceptibility to disease. PFOS-induced changes in miR-451, miR-23a, and miR-24 have likewise been linked to heightened inflammatory states, highlighting a broader impact on immune regulation [94].

A parallel line of evidence underscores that PFOS-induced neurotoxicity hinges on ncRNA dysregulation, particularly in the context of brain-derived neurotrophic factor (BDNF) signaling [95]. BDNF is a critical neurotrophic factor involved in neuronal development, synaptic plasticity, and survival [96,97,98]. Li and co-workers (2015) [95] demonstrated that PFOS disrupts BDNF–ERK–CREB signaling by altering the balance of two key miRNAs that modulate BDNF expression: miR-16 and miR-22. Under normal conditions, miR-16 represses BDNF by binding to its mRNA; however, PFOS exposure decreases miR-16 levels, a change that might be expected to elevate BDNF as a compensatory mechanism but ultimately does not rescue BDNF’s neuroprotective functions due to concurrent upregulation of other inhibitory factors [95]. In parallel, miR-22, which also targets BDNF mRNA and suppresses its protein expression, is significantly upregulated by PFOS, causing a pronounced decrease in BDNF levels and further contributing to neuronal vulnerability [95]. Despite elevated CREB (cAMP-responsive element binding protein) phosphorylation (an event usually indicative of enhanced BDNF transcription), PFOS-exposed cells exhibit reduced BDNF mRNA and protein levels, suggesting a disconnect in the signaling cascade. This disruption appears to stem from the combined modulation of miR-16 and miR-22, revealing a complex miRNA network that skews toward neurotoxicity by diminishing BDNF-mediated neuronal support. Mechanistically, such miRNA-driven changes can propagate through broader epigenetic networks, ultimately impacting neuronal survival and synaptic function and potentially contributing to neurodevelopmental deficits [95]. These findings, even though solely derived in vitro and lacking realistic exposure kinetics, have not only established miRNAs as key regulators of PFOS-induced neurotoxicity but also point to their potential utility as biomarkers and therapeutic targets to mitigate PFAS-related neurological risk [95].

In addition to their direct roles in transcriptional and post-transcriptional regulation, lncRNAs can further influence miRNA function by acting as competing endogenous RNAs, sequestering these small RNAs and thereby modulating a wide array of downstream gene targets [99,100]. Disruption of such “sponge-like” interactions under PFOS exposure could compromise cell cycle control, stress responses, and chromatin remodeling. Indeed, PFOS-induced differential methylation of lncRNAs linked to CDK4, p21, p27, and p53 underscores the breadth of PFAS-related epigenetic perturbations, even in cases where the promoters of these cell cycle genes remain unaffected [89]. In parallel, PFAS exposure has been associated with altered estrogen receptor (ER) signaling through differential methylation of lncRNAs that modulate ESR1 and ESR2, as well as downstream targets such as GREB1 and RERG, which govern estrogen-dependent cell proliferation [89]. Given that aberrant estrogen signaling is closely tied to hormone-related cancers, these findings reinforce the notion that PFAS poses multifaceted health risks across multiple tissues and organ systems.

Thus, PFAS, particularly PFOS, can orchestrate complex and persistent epigenetic shifts involving lncRNAs and miRNAs, driving dysregulated inflammatory responses, placental dysfunction, carcinogenic processes, and neurotoxicity. The MEG3/miR-770/PTX3 axis in the placenta exemplifies a precise mechanism by which PFOS-induced hypermethylation of MEG3 and subsequent disruption of miR-770 lead to PTX3 overexpression, inhibiting trophoblast cell growth and impairing placental development [87]. Meanwhile, BDNF–ERK–CREB dysregulation in neuronal cells, mediated by altered miRNA expression, reveals a parallel route for PFOS-driven harm in the central nervous system [95]. These mechanistic insights complement epigenome-wide data showing hypermethylation across cancer-related lncRNAs, which may contribute to increased malignancy risk. From a public health standpoint, identifying epigenetic signatures, be they differentially methylated lncRNAs such as GACAT3, CASC2, DELEC1, and LCIIAR or miRNAs such as miR-22 and miR-19a-3p, could enable early biomarker-driven assessment of PFAS exposure risks. Equally promising, therapeutic strategies targeting DNA methylation patterns or modulating ncRNA levels may hold promise for mitigating PFAS-induced pathologies.

## 3. Prenatal PFAS Exposure and Epigenetic Programming

### 3.1. Epigenetics and Development

In mammalian development, the establishment and precise modulation of the epigenome is critical for orchestrating cellular differentiation, lineage commitment, and the maintenance of genomic imprinting [101,102,103]. Within the framework of the Developmental Origins of Health and Disease (DOHaD) hypothesis, a conceptual model that elucidates how early-life environmental exposures can induce stable epigenetic alterations predisposing individuals to metabolic, cardiovascular, and neurodevelopmental disorders later in life [104,105,106]. PFAS can potentially disrupt DNA methylation, histone modification dynamics, and non-coding RNA expression, thereby compromising the precise epigenetic programming required for normal development. Of particular significance are the two waves of epigenetic reprogramming during embryogenesis, which reset and re-establish parent-of-origin methylation patterns critical for imprinting, transposon silencing, and lineage specification [43,47,49,107,108]. Recent advancements in high-resolution and single-cell epigenomic profiling include techniques such as chromatin immunoprecipitation sequencing (ChIP-seq), ATAC-seq, reduced representation bisulfite sequencing (RRBS), and nascent transcript profiling, which have significantly refined our understanding of these processes. However, while these methodologies offer unprecedented detail, they are not without limitations. Challenges related to resolution limits, reproducibility across platforms, and potential biases (e.g., antibody specificity in ChIP-seq) warrant careful interpretation of the data [12,50].

The vulnerability of imprinted genes, such as *MEST* and *IGF2*, which play critical roles in trophoblast differentiation and embryonic growth, respectively, exhibits disrupted methylation patterns following PFAS exposure, further exemplifying the intricate interplay between environmental insults and developmental epigenetics [33,34,109]. *IGF2* is one of the most extensively characterized imprinted genes. It is expressed predominantly from the paternal allele, with its proper dosage maintained by differential DNA methylation at its imprinting control regions (ICRs) and further regulated by coordinated histone modifications and non-coding RNAs [110,111]. Critically, disruptions in one-carbon metabolism, which is integral to the synthesis of methyl donors such as SAM, can impair the activities of DNMT and TET enzymes, thereby compromising the establishment and maintenance of methylation marks at IGF2’s ICRs. Such perturbations can lead to aberrant imprinting and dysregulated IGF2 expression, with significant implications for fetal growth and long-term health outcomes [33,34,109]. Similarly, MEST, predominantly expressed in mesoderm-derived tissues and the placenta, exhibits a variable imprinting pattern that renders it particularly sensitive to environmental perturbations. Altered epigenetic regulation of MEST has been implicated in abnormal fetal growth trajectories and may predispose individuals to metabolic dysfunction later in life [112,113].

Similarly, prenatal exposure to PFAS can induce sex-specific epigenetic modifications that may alter developmental trajectories in a divergent manner between male and female fetuses. Several epidemiological studies have reported that DNA methylation profiles in umbilical cord blood differ by sex in relation to maternal PFAS exposure. For example, work by Hu and colleagues (2023) and Miura and colleagues (2018) identified distinct sets of differentially methylated positions in male versus female newborns [69,114,115]; in males, alterations were often observed in genes related to immune regulation and reproductive functions, whereas in females, the changes tended to involve genes associated with metabolic regulation and neurodevelopment [43]. Mechanistically, these differences may also be explained by perturbations in one-carbon metabolism in DNA methylation as well as by differential modulation of DNMTs and TET enzymes by endogenous sex hormones [116]. Furthermore, animal studies have demonstrated that PFAS exposure can affect nuclear receptor signaling (notably via peroxisome proliferator-activated receptors or PPARs) in a sex-dependent fashion, thereby influencing chromatin remodeling and subsequent gene expression patterns.

Similarly, prenatal exposure to PFAS can induce sex-specific epigenetic modifications that may alter developmental trajectories in divergent ways between male and female fetuses. Several population-based studies have reported that DNA methylation profiles in umbilical cord blood differ by sex in relation to maternal PFAS exposure. For instance, work by Leung and co-workers (2018) [117] and Miura and co-workers (2018) [69] dentified distinct sets of differentially methylated positions in male versus female newborns; in males, alterations were often observed in genes related to immune regulation and reproductive functions, whereas in females, the changes tended to involve genes associated with metabolic regulation and neurodevelopment [43]. Beyond this, PFAS exposure is intricately linked to endocrine disruption, particularly within pathways governing thyroid hormone homeostasis and reproductive function [118,119,120]. Epigenetic alterations in genes encoding thyroid hormone receptors and co-regulators and those involved in steroidogenesis may disrupt hormone production, transport, and signaling, thereby affecting fetal growth and neurodevelopment. Mechanistically, these sex-specific differences may be further explained by perturbations in one-carbon metabolism affecting DNA methylation and differential modulation of DNMTs and TET enzymes by endogenous sex hormones [116]. Furthermore, animal studies have demonstrated that PFAS exposure can affect nuclear receptor signaling, notably via PPARs in a sex-dependent fashion, thereby influencing chromatin remodeling and subsequent gene expression patterns.

Additionally, several studies have documented congener-specific patterns of epigenetic dysregulation that differ by fetal sex. For instance, Everson and co-workers (2025) [17] reported that female fetuses exhibited more differentially methylated loci in response to PFHxS and PFOS exposure. In contrast, Wang and co-workers (2023) [121] observed that male fetuses were more susceptible to PFAS-induced alterations in LINE-1 methylation. Complementing these observations, Petroff and co-workers (2023) [20] demonstrated distinct DNA methylation and hydroxymethylation patterns between male and female fetuses: PFHxS exerted a more pronounced effect on males with 81 significant CpG sites compared to 17 in females, while PFOS predominantly impacted females, affecting 78 sites versus 10 in males. Similar sex-specific associations were observed for other congeners, including PFNA, PFDA, and PFUnDA, highlighting differential susceptibilities to epigenetic alterations. Notably, genes integral to spermatogenesis (e.g., *SPATA4*, *RNF5*, and *RNF5P1*) were differentially methylated in males, whereas females exhibited significant epigenetic changes in genes involved in metabolic regulation and neurodevelopment (e.g., *SPATS2L* and *RAP1GAP2*) [16,17,20,121].

Complementing these molecular insights, human cohort studies have further substantiated the epigenetic impact of prenatal PFAS exposure on developmental health. Analyses within the Project Viva cohort identified 435 differentially methylated CpG sites associated with PFAS exposure, with congener-specific trends such as predominant hypomethylation linked to PFNA [61]. In parallel, epigenome-wide association studies in Japanese cohorts uncovered approximately 854 significant CpG sites, highlighting specific loci directly relevant to developmental disorders. Among these, cg16242615 (mapped to ZBTB7A) has been associated with neurodevelopmental disorders [122], while cg21876869 in the intergenic region of USP2-AS1 has implications in oncogenesis [123]. Additional sites, such as cg00173435 (mapped to TCP11L2) and cg18901140, located near NTN1, have been linked to muscle cell migration, differentiation [124], and processes critical for nervous system development, angiogenesis, and organogenesis [125,126,127,128]. Furthermore, recent work by Petroff and colleagues (2023) [20] expanded these observations by identifying approximately 5036 5-methylcytosine (5-mC)-specific and 13,376 5-hydroxymethylcytosine (5-hmC)-specific CpG sites across eight PFAS compounds, demonstrating that first-trimester exposures are correlated with DMRs in genes implicated in autophagy and neurodevelopment [20].

The translational implications of PFAS-induced epigenetic alterations are profound. Changes in the epigenome during critical developmental windows affect placental function and fetal growth and may establish an “epigenetic memory” that predisposes individuals to a range of long-term health disorders. For example, dysregulation of genes involved in brain development could elevate the risk for neurodevelopmental conditions such as attention-deficit/hyperactivity disorder (ADHD) or autism spectrum disorders. In contrast, perturbations in metabolic gene networks may increase susceptibility to obesity, type 2 diabetes, and cardiovascular disease later in life [121,129]. Critically, these immediate epigenetic consequences raise critical questions regarding the potential for transgenerational inheritance. Should PFAS-induced modifications evade the reprogramming events in the germline, they might predispose subsequent generations to similar developmental and metabolic disorders, amplifying long-term public health concerns [102,103,130,131]. This dual impact underscores the urgency of integrating multi-generational study designs and animal models to elucidate the precise molecular pathways involved, ensuring that both immediate and long-term health implications are adequately addressed.

The translational implications of PFAS-induced epigenetic alterations are profound. Changes in the epigenome during critical windows of development affect placental function and fetal growth and may also establish an “epigenetic memory” that predisposes individuals to a range of long-term health disorders. For instance, dysregulation of genes governing brain development could elevate the risk for neurodevelopmental conditions such as ADHD [132] and autism spectrum disorders [133]. In contrast, disturbances in metabolic gene networks may increase the likelihood of obesity, type 2 diabetes, and cardiovascular disease later in life [121,129]. Moreover, emerging evidence indicates that some PFAS-induced epigenetic marks may evade the extensive reprogramming processes of the germline, potentially persisting across generations and predisposing subsequent cohorts to developmental and metabolic disorders [102,103,130,131]. The intersection of these molecular alterations with neurodevelopmental outcomes is further highlighted by observations of delayed language acquisition and motor coordination deficits [134,135].

#### Epigenetic Changes in the Placenta and Embryos

Within placental and embryonic tissues, PFAS exposure is increasingly recognized as a potent disruptor of the intricate epigenetic programming necessary for normal prenatal development [17,121]. These disruptions coincide with periods of intense epigenetic reprogramming. Consequently, these ubiquitous compounds readily cross the placenta and accumulate in fetal tissues, accumulating in the placenta, a critical organ mediating nutrient transport, hormonal signaling, and immune regulation during gestation [3,130,136,137]. Emerging evidence indicates that PFAS exposure can simultaneously perturb multiple layers of epigenetic regulation in the placenta, which orchestrate gene expression patterns essential for fetal growth and long-term health outcomes [18,130].

Recent human placental studies provide compelling insights into PFAS-induced DNA methylation changes. In an epigenome-wide association study, Everson and colleagues (2025) [17] evaluated 17 PFAS compounds in placental tissues and detected five compounds (including PFHxS, PFOS, PFOA, PFNA, and PFDA) in over 70% of samples. Notably, PFHxS was significantly associated with altered methylation at 11 loci, many of which mapped to genes such as XKR6, NAV2, and KCNQ3 genes implicated in lipid metabolism, hormone regulation, and placental development [17]. Complementary findings by Wang and co-workers (2023) [121] revealed that PFAS exposure correlates with hypomethylation of key regulatory regions in genes such as IGF2, NR3C1 (encoding the glucocorticoid receptor), and the repetitive element LINE-1. Specifically, PFOS was inversely correlated with LINE-1 methylation, a marker of genomic stability, while PFOA-dominated mixtures were linked to reduced methylation at NR3C1, potentially compromising the fetal stress response [17,121].

Animal models have further illuminated the mechanistic basis of these epigenetic perturbations in the placenta. Hallberg and co-workers (2022) [129] demonstrated that exposure to PFHxS during the in vitro maturation (IVM) of bovine cumulus-oocyte complexes results in persistent alterations in DNA methylation and gene expression that extend into the blastocyst stage. Their analysis identified 668 DMRs, predominantly enriched in CpG islands, overlapping significantly with differentially expressed genes (DEGs) [129]. Notably, approximately half of these loci exhibited an inverse correlation between methylation and gene expression, suggesting a direct regulatory influence of PFAS on gene activity. Pathway analyses further revealed that PFHxS exposure induced transcriptional changes in genes involved in oxidative stress response, lipid metabolism, and hormonal signaling, including the ATM, TP53, TGF-β, PPARγ, and ER pathways [129]. These findings provide a mechanistic framework in which PFAS exposure may initiate oxidative stress and disrupt endocrine signaling, thereby impairing oocyte maturation and early embryonic development. Although the concentrations used in these animal studies are higher than those typically encountered by the general population, they are nevertheless relevant for highly exposed cohorts, such as industrial workers or populations residing near contamination sites [121].

A critical evaluation of the methodologies employed in these studies reveals both strengths and limitations. Epigenome-wide association studies, such as those conducted by Everson and co-workers (2025), offer comprehensive profiling of methylation changes; however, issues related to sample heterogeneity and limited resolution at low-exposure levels remain [17]. Similarly, while in vitro models such as Hallberg and co-workers (2022) provide valuable mechanistic insights, their extrapolation to human exposure scenarios is tempered by interspecies biological differences and exposure dose discrepancies. Consolidating human and animal studies, evidence suggests that PFAS exposure during critical periods of placental and embryonic development induces a spectrum of epigenetic modifications with lasting consequences [17,121,129]. These modifications, including changes in DNA methylation, histone modification, and non-coding RNA expression, disrupt key regulatory pathways involved in growth, metabolism, and hormonal signaling. While current research provides a strong foundation for understanding these effects, further studies employing integrative and standardized approaches are needed to fully elucidate the mechanisms, assess dose-response relationships, and explore sex-specific vulnerabilities [3,130]. In doing so, the field will be better positioned to inform public health policies and develop targeted interventions, such as nutritional strategies to enhance methyl donor availability, to mitigate the long-term risks associated with PFAS exposure.

## 4. Health Risks Associated with PFAS-Induced Epigenetic Changes In Utero

A growing body of evidence from epidemiological investigations, animal models, and in vitro studies suggests that PFAS ranging from legacy compounds such as PFOA and PFOS to emerging short-chain analogs disrupt normal gestational physiology, thereby contributing to adverse outcomes, including preeclampsia, low birth weight, neurodevelopmental impairments, cardio-metabolic diseases, and immune dysfunction, central around epigenetic dysregulation that perturb gene regulatory networks essential for placental function, fetal growth, and metabolic homeostasis [109,138,139]. For instance, while several studies have linked elevated maternal PFAS levels with late-onset preeclampsia, others highlight dose-response relationships that manifest in diminished birth weights and impaired trophoblast migration through disrupted signaling pathways and epigenetic reprogramming. Similarly, neurodevelopmental outcomes appear to be modulated by PFAS-induced changes in DNA methylation and histone acetylation patterns in the developing brain, with emerging multi-omics approaches beginning to unravel cell-type-specific vulnerabilities. In addition, the dysregulation of metabolic gene networks, which is evident from the altered expression of lipid-handling genes and insulin signaling mediators, further implicates epigenetic modifications in the developmental origins of cardiometabolic diseases. Despite these compelling insights, controversies persist regarding exposure timing, dose dependency, and the potential for intergenerational transmission of epigenetic marks, all of which underscore the inherent complexity of PFAS toxicity.

### 4.1. An Integrated Epidemiological and Mechanistic Perspective of Epigenetic Dysregulation of Maternal PFAS Exposure and Low Birth Weight

Maternal exposure to PFAS is a significant contributor to low birth weight (LBW), defined as a newborn weight of less than 2500 g, a critical public health concern due to its association with increased risks of both short- and long-term adverse health outcomes [140]. Epidemiological studies have consistently linked in utero PFAS exposure with impaired fetal growth. For instance, Wikström and colleagues (2020) [141]. conducted a robust longitudinal cohort study involving 2355 pregnant women, assessing eight PFAS compounds including PFOS, PFOA, PFHxS, PFNA, PFDA, PFUnDA, PFHpA, and PFDoDA, and identified significant inverse associations between maternal serum levels of PFOS, PFOA, PFNA, PFDA, and PFUnDA and infant birthweight, as recorded in the Swedish Medical Birth Register [141]. Complementing these findings, Tian and co-workers (2023) analyzed 15 PFAS in maternal blood samples collected shortly before delivery and reported a heightened risk of LBW, mainly linked to PFOA exposure [142]. Nevertheless, the exposure assessment was limited to late gestation, potentially missing earlier critical windows, and the study’s observational design precludes causal inference.

Mechanistically, PFAS may compromise fetal growth, resulting in LBW through multiple pathways. After traversing the placental barrier, inducing placental insufficiency, disrupting fetal circulation, and interfering with endocrine regulation essential for pregnancy maintenance [143,144], emerging research implicates epigenetic modifications as a key underlying mediator leading the LBW process. Steenland and co-workers (2018) [145], in a meta-analysis of nearly 13,000 births, documented a modest inverse relationship between PFOA levels and birthweight with an approximate reduction of 10.5 g per 1 ng/mL increase in PFOA highlighting that the timing of exposure measurement (early versus late gestation) may modulate this association through physiologic changes during pregnancy [145]. Still, variability among studies and the modest effect size limit causal interpretations. Building on these observations, Wright and colleagues (2023) reported that PFNA exposure was associated with a more pronounced birth weight deficit, approximately 32.9 g per natural log unit increase in concentration, with sensitivity analyses suggesting cumulative exposure effects or periods of heightened fetal vulnerability [146]. They further suggest the role of PFAS-induced epigenetic alterations, suggesting how to compromise the regulation of genes pivotal for placental and fetal development, although direct epigenetic endpoints remain underexplored.

Consequently, direct assessments of epigenetic modifications have begun to elucidate these mechanisms. Ku and colleagues (2022) examined prenatal exposure to PFOS in a Taiwanese cohort and employed ultra-performance liquid chromatography-tandem mass spectrometry (UPLC–MS/MS) alongside pyrosequencing of the MEST imprinted gene. Their results demonstrated that higher PFOS levels were significantly associated with hypomethylation at multiple CpG sites, and mediation analyses revealed that while PFOS exerted direct deleterious effects on birth weight, the concurrent epigenetic changes in MEST appeared to have a partial compensatory role, an effect that was notably more pronounced in female infants [109]. Similarly, Wang and colleagues (2023) [121] focused on placental tissue from 180 pregnant women and found significant PFAS-associated alterations in DNA methylation of key growth-regulatory genes such as IGF2, NR3C1, and the repetitive element LINE-1. The observed inverse correlation between PFOS and LINE-1 methylation, along with associations between PFAS mixtures (with a substantial contribution from PFOA) and reduced head circumference, underscores the placenta’s role as a critical mediator of PFAS-induced epigenetic dysregulation and fetal growth impairment [121]. Further supporting the interplay between endocrine disruption and epigenetic regulation, Qian Yao and colleagues (2021) explored the endocrine milieu in a prospective birth cohort. By documenting alterations in placental P450 aromatase and cord serum levels of estradiol and testosterone coupled with a trend toward lower birthweight, they provided indirect evidence that PFAS exposure may initiate epigenetic perturbations, a hypothesis bolstered by the use of paternal PFAS exposure as a negative control to minimize confounding by shared familial factors [147]. While this indirect approach provides valuable insights, it may not entirely establish causality or identify specific epigenetic changes, even when using paternal exposure as a negative control to minimize shared confounding factors.

In synthesis, these studies collectively portray a complex landscape in which maternal PFAS exposure influences fetal growth through both direct toxic effects and indirect epigenetic mechanisms, resulting in LBW [121,145,146]. Emerging trends in this field advocate a shift toward integrating mechanistic epigenetic endpoints with traditional epidemiological assessments. Such integration not only refines our understanding of PFAS-induced fetal growth impairment but also provides a framework for developing targeted interventions and regulatory policies. Ultimately, advancing our understanding in this area is critical for mitigating the developmental origins of health and disease in populations exposed to these persistent environmental toxicants.

### 4.2. Molecular Mechanisms and Neurodevelopmental Impact of Maternal PFAS Epigenetic Disruption

The fetal brain, characterized by rapid growth and differentiation, is particularly susceptible to toxicants, as PFAS cross the placental barrier, accumulate in fetal tissues, and persist postnatally [148,149,150], leading to disorders encompassing a spectrum of conditions that impair neurological function and cognitive outcomes, thereby affecting learning and memory [151]. Mechanistically, PFAS disrupts the regulation of key epigenetic processes that are essential for orchestrating neurogenesis, synaptic plasticity, and the establishment of neuronal connectivity [152,153,154]. For instance, PFAS-induced hypomethylation in the promoter regions of neurodevelopmental genes has been associated with aberrant gene expression and impaired neuronal differentiation in human-induced pluripotent stem cell (hiPSC)-derived cortical neurons [153,155]. Quantitative analyses further reveal that even low-level exposures such as 0.4 ppb PFOA can significantly reduce neurite outgrowth and alter DNA methylation at critical CpG sites regulating tau protein expression, suggesting a dose-response relationship that warrants further exploration [148,150,155].

Integrative multi-omics approaches have been instrumental in unraveling the molecular cascades underpinning PFAS-mediated neurotoxicity. Coupled epigenomic, transcriptomic, and proteomic data analyses have identified disrupted Neurodevelopmental networks involving oxidative stress, mitochondrial dysfunction, and perturbations in PPAR signaling pathways [148,150,153]. In zebrafish models, PFOS exposure has been linked to oxidative stress and altered calcium signaling, accompanied by modifications in histone marks such as H3K27 acetylation that correlate with behavioral deficits [148,150]. However, these neurobehavioral outcomes need careful interpretation, as species-specific developmental differences and high exposure levels limit direct applicability to humans. Although data on specific histone acetylation and methylation events remain limited, preliminary findings suggest that PFAS-induced oxidative stress and thyroid hormone disruption may remodel chromatin accessibility at loci critical for neuronal differentiation [133,153]. Thyroid hormone dysregulation, a well-documented consequence of PFAS exposure, further exacerbates these epigenetic disturbances by modulating hormone-responsive elements within neurodevelopmental gene networks [152,156]. Notably, sex-specific differences in neurodevelopmental consequences emerged, with male offspring often exhibiting more pronounced reductions in DNA methylation at key genomic loci, potentially due to differential regulation of hormone-responsive genes [20,43,134,152,157], while female offspring may display unique patterns of epigenetic regulation that confer either heightened vulnerability or unexpected resilience [133,158]. Maternal factors, including age, diet, and parity, further modulate these outcomes [134,156].

Complementing these mechanistic insights, human epidemiological and animal studies have provided a multifaceted, albeit sometimes contradictory, perspective on the neurodevelopmental impacts of prenatal PFAS exposure. PFAS compounds in utero potentially disrupt the blood–brain barrier by virtue of their ability to cross the placental barrier [156] and their links to impairments in cognitive function, motor skills, and social behavior [156,159,160]. For example, Niu and co-workers (2019) [161] examined a cohort of 981 pregnant women, measuring PFAS compounds, including PFOS, PFOA, PFHxS, PFNA, PFDA, PFUnDA, PFDoA, and PFTrDA, in maternal plasma at 12–16 weeks of gestation. Using the Ages and Stages Questionnaires (ASQ) to assess neuropsychological development in 661 mother–infant pairs, the study found that elevated maternal PFAS concentrations were associated with deficits in personal–social skills at four years of age, with PFTrDA specifically linked to an increased risk of communication problems, while no significant associations were observed for gross motor function, fine motor function, or problem-solving ability [161]. However, the reliance on parent-reported developmental assessments and the attrition of the cohort (661 of 981 pairs analyzed) may introduce bias, and this observational study cannot establish a causal link. In a complementary investigation, the Health Outcomes and Measures of the Environment (HOME) study by Vuong and co-workers (2021) [134] assessed 242 mother–child pairs from Greater Cincinnati, Ohio. Maternal sera, collected at 16 and 26 weeks of gestation and at delivery, were analyzed for PFOA, PFOS, PFHxS, and PFNA, while child behavior was evaluated using the Behavioral Assessment System for Children 2 (BASC-2) and the Diagnostic Interview Schedule for Children–Young Child version (DISC-YC), reported that prenatal exposures to PFOS and PFNA were associated with increased hyperactivity and ADHD-like behaviors, whereas PFHxS exposure correlated with internalizing problems, including anxiety, depression, and somatization [134]. Nevertheless, the relatively small sample size and regional homogeneity of this cohort limit the generalizability of the findings, and the use of caregiver-reported behavioral measures may be subject to reporting bias. When integrated with molecular data, these epidemiological findings underscore the complexity of PFAS toxicokinetics and highlight the interplay between epigenetic modifications and neurodevelopmental outcomes.

Collectively, the convergence of mechanistic and epidemiological data suggests that PFAS-induced epigenetic reprogramming plays a pivotal role in mediating neurodevelopmental toxicity. The observed reversibility of some epigenetic modifications further opens promising avenues for therapeutic intervention using epigenetic modulators, aligning with the broader paradigm shift toward precision medicine and targeted risk assessment [28,150,153,154,162]. Future research employing standardized, longitudinal study designs that integrate robust epigenetic endpoints is essential for resolving current controversies and advancing our understanding of PFAS-related neurotoxicity.

### 4.3. PFAS-Induced Epigenetic Reprogramming and Metabolic Dysregulation In Utero

Emerging evidence suggests that even chronic low-dose exposure to PFAS such as PFOA and PFOS leads to bioaccumulation and prolonged metabolic toxicity through aberrant DNA methylation of metabolic genes and altering histone marks, which compromises critical pathways such as insulin signaling and lipid metabolism [56]. Prenatal exposure has been implicated in the developmental programming of metabolic disorders, including obesity, type 2 diabetes (T2D), and cardiovascular diseases [163,164,165,166]. Crucially, both direct fetal exposure and parental germline modifications suggest that even minimal PFAS doses can exert far-reaching, multigenerational impacts on metabolic homeostasis [163,164,165,166,167,168,169].

Recent studies employing animal models have revealed that even newer alternative short-chain PFAS analogs, such as perfluorobutane sulfonate (PFBS), elicit profound metabolic reprogramming. For instance, Meng et al. (2023) demonstrated that although classical biochemical markers remained largely unchanged in PFBS-exposed rats, significant alterations were observed in genes regulating metabolic pathways, including PPAR signaling, cytochrome P450-mediated xenobiotic metabolism, amino acid turnover, and unsaturated fatty acid biosynthesis [170,171]. Nonetheless, these rodent studies arise from limited controlled experiments, and the metabolic differences between species restrict direct comparisons to human populations. The downregulation of key lipid-handling genes such as Fabp4, Fabp2, and stearoyl-CoA desaturase (Scd) was accompanied by notable shifts in lipid metabolites, suggesting that these transcriptional changes likely stem from upstream epigenetic modifications [163,165]. Beyond alterations in transcriptomic reprogramming, PFAS-induced epigenetic metabolic regulation was performed by Ho and colleagues (2023), employing whole-genome bisulfite sequencing (WGBS) in a murine model that identified 272 DMRs in the fetal liver following PFOS exposure, with these regions enriched in genes governing fatty acid and glucose metabolism as well as inflammatory responses [164,171]. Notably, concomitant changes in histone modifications, such as increased H3K9 acetylation and aberrant H3K27 trimethylation, suggest that PFAS exposure dynamically alters the recruitment of transcription factors to regulatory regions [163]. Additionally, data further implicate the dysregulation of non-coding RNAs; while several studies have focused on microRNA perturbations (e.g., miR-33, miR-122, and miR-375) [163,172], and recent high-throughput RNA sequencing efforts have identified lncRNAs as critical scaffolds and regulators in the orchestration of metabolic gene networks [87,173].

Population-based studies further bolster the mechanistic insights gained from experimental models. Several studies have linked prenatal PFAS exposure to increased childhood adiposity, elevated body mass index, and dysregulated lipid profiles [166,172,174]. However, translating these findings to human health is not without challenges, as species-specific differences in PFAS metabolism and the presence of confounding environmental factors complicate direct extrapolations [157,159]. Although, collectively, and as acknowledged in the studies [163,166] these observational cohorts while providing valuable associations are constrained by heterogeneity in PFAS congener selection and analytical methods, variability in sampling windows (maternal serum versus cord blood), incomplete adjustment for diet, socioeconomic and co-exposure confounders, and limited longitudinal follow-up, all of which undermine causal inference and the broader generalizability.

The complexity of PFAS-induced metabolic epigenetic reprogramming is further underscored by the influence of dose–response relationships and temporal dynamics. Differences in exposure duration, critical gestational window timing, and PFAS species, e.g., legacy compounds such as PFOA and PFOS versus newer short-chain analogs, can differentially impact the epigenetic backdrop, as reported in Yu et al. [72,175]. For example, Starling and colleagues (2020) observed that prenatal exposure to PFOA and PFOS not only temporally altered DNA methylation patterns at key metabolic regulators (e.g., loci controlling cholesterol metabolism and insulin signaling) but also correlated with clinical markers of intrauterine growth restriction, such as low birth weight [72]. Hypomethylation of IGF2 provided mechanistic support for PFAS influence on metabolic health via epigenetic regulation [33,72]. That said, the study is observational, meaning its findings might vary based on the specific population and could be affected by factors we have not measured and by past exposure experiences. Other DMRs were detected for multiple PFAS, with genes implicated in lipid metabolism (PON1, CIDEB, NR1H2), growth regulation (RPTOR), and immune activity (RNF39, RASL11B) [72,166]. These findings highlight the context-dependent nature of PFAS toxicity and underscore the necessity for a more granular understanding of exposure kinetics [169].

Additionally, the dysregulation of IGF2, often resulting from loss of imprinting (LOI) or other epigenetic modifications, has been suggested to contribute to PFAS-induced metabolic and Epigenetic anomalies in later life [73,176]. Studies have observed associations between elevated IGF2 expression and insulin resistance in specific tissues, suggesting that altered IGF2 signaling could play a role in the etiology of type 2 diabetes and related metabolic disorders [177,178]. Consistent with these observations, genetic studies have revealed that polymorphisms in the IGF2 gene are associated with increased body weight, adiposity, and visceral fat accumulation, further linking IGF2 to obesity and metabolic syndrome [177]. Importantly, circulating IGF2 levels are often elevated in obesity but can be reduced with weight loss, reinforcing its metabolic role. The IR-A isoform, which binds IGF2, is upregulated in insulin-resistant states, potentially increasing IGF2-driven metabolic dysregulation [177]. Keating and El-Osta (2015) highlight IGF2 promoter hypermethylation in response to maternal low-protein diets, which increases IGF2 expression and may contribute to fetal programming of metabolic dysregulation [179]. Furthermore, differential methylation of IGF2 has been linked to offspring born to mothers with impaired glucose tolerance, reinforcing its role in long-term metabolic outcomes [177].

At the tissue level, epigenetic modifications of IGF2 appear to be highly context-dependent, as indicated by H3K4me3 enrichment at the IGF2 promoter in vascular endothelial cells but not in monocytes, suggesting that IGF2-driven metabolic outcomes are cell-type specific [177]. Beyond its metabolic functions, IGF2 is a potent mitogen, influencing cell proliferation, differentiation, and organogenesis, which may explain why its dysregulation is often implicated in oncogenic processes as well as metabolic disorders [177]. However, in many experimental models, the primary perturbations linked to IGF2 dysregulation manifest as aberrant growth and developmental defects rather than isolated metabolic dysfunction [179,180,181]. This is consistent with findings that IGF2 loss of imprinting is commonly observed in tumors, suggesting a dual role in both metabolic and proliferative pathways [177]. Importantly, the metabolic consequences of IGF2 misexpression appear to be highly dependent on developmental stage, hormonal environment, and external metabolic factors, as illustrated by the differential methylation of IGF-related genes in response to maternal diet and metabolic status. For example, miR-483, encoded within the IGF2 locus, inhibits adipogenesis and myogenesis, while miR-675, derived from H19, which has been implicated in PFAS-induced methylation and adipocyte differentiation and insulin sensitivity, further highlights the complex regulatory network governing IGF2 function [73,177]. This suggests that the epigenetic imprinting of IGF2 may serve as a critical determinant of whether its dysregulation leads to metabolic disturbances, developmental abnormalities, or both [176,177].

The intergenerational transmission of PFAS-induced epigenetic changes represents a particularly compelling aspect of this emerging field. Maxwell and co-workers (2024) reported that paternal PFAS exposure resulted in widespread epigenetic alterations in sperm, with thousands of DMRs identified in promoters of genes central to lipid and cholesterol metabolism [167,168]. These epigenetic modifications were mirrored by significant transcriptomic changes in the liver and adipose tissues of offspring, further implicating disrupted epigenetic inheritance in the pathogenesis of metabolic disorders [163,168]. Complementary evidence from Drosophila models underscores the conservation of these mechanisms, demonstrating that multigenerational PFAS exposure disrupts key regulators such as Sirt1 and Polycomb group proteins, ultimately affecting metabolic gene expression [87,168].

Thus, the current body of evidence elucidates a complex interplay between PFAS-induced epigenetic reprogramming and metabolic dysregulation. The convergence of altered epigenetic dynamics forms a mechanistic basis for the observed transcriptional and metabolic changes that predispose individuals to obesity and T2D [163,164,165,172]. Despite these advances, significant gaps remain, particularly regarding dose-response relationships, temporal dynamics, and intergenerational transmission of these effects [167,169]. Addressing these challenges is paramount for refining our understanding of PFAS toxicity and for informing regulatory policies aimed at mitigating the long-term health impacts of these pervasive environmental contaminants [166,175].

### 4.4. Cardiovascular and Cardiometabolic Diseases

PFAS exposure is a significant risk factor for a spectrum of cardiometabolic diseases, including dyslipidemia, obesity, diabetes, and cardiovascular diseases, with evidence implicating both metabolic dysregulation and epigenetic reprogramming in the mediation of their pathogenicity [9,182,183]. At the metabolic level, PFAS exposures have been consistently shown to interfere with lipid homeostasis. Several PFAS compounds activate peroxisome proliferator-activated receptor α (PPARα), thereby enhancing fatty acid oxidation and, paradoxically, reducing serum cholesterol levels [182,184]. Yet, despite these observations, the impact on glucolipid metabolism remains ambiguous. Some studies suggest that PFAS inhibits the tyrosine phosphorylation of insulin receptor substrates, thereby disrupting insulin signaling and diminishing insulin sensitivity through subsequent protein kinase activation. Notably, the structural similarity of PFAS to fatty acids may upregulate genes involved in fatty acid oxidation, promoting oxidative stress and exacerbating insulin resistance [185].

Complementary to these metabolic perturbations, epidemiological investigations have provided critical insights into the life-course impact of PFAS exposure. For example, analysis of the follow-up of the HOME study over 12 years revealed that high serum concentrations of PFOA and PFHXS during gestation were positively associated with biomarkers indicative of cardiometabolic risk in children [186,187]. While this single-cohort observational follow-up presents valuable insights, it is important to note that it may be influenced by potential attrition bias. As such, we cannot definitively establish causality, and the findings might show traits specific to the HOME study population. These findings underscore the notion that early-life exposure to PFAS can predispose individuals to later-life metabolic and cardiovascular dysfunction.

At the molecular level, a growing body of research now implicates PFAS-induced epigenetic reprogramming as a critical link between environmental exposure and altered metabolic and vascular function. Mechanistic studies in vitro have demonstrated that exposure to legacy PFAS such as PFOA and PFOS can trigger a cascade of intracellular events, culminating in altered gene expression. For instance, Caroccia et al. (2023) reported that PFAS exposure in human adrenocortical cells induces a concentration-dependent upregulation of the aldosterone synthase gene (*CYP11B2*), an effect driven by increased mitochondrial reactive oxygen species (ROS) that may establish an epigenetic “memory” through enhanced accessibility of key regulatory regions [188,189,190]. It is important to mention that this is also an in vitro study.

Epidemiological studies further corroborate these mechanistic insights. Gump et al. (2023) found that elevated serum PFAS levels, especially in conjunction with lead exposure, were associated with heightened vascular reactivity and increased carotid intima-media thickness (cIMT) in children, suggesting that epigenetic modifications affecting lipid metabolism and insulin signaling underlie these vascular alterations [191]. Nonetheless, the concurrent exposure to lead and the study’s cross-sectional nature complicates the attribution of vascular changes specifically to PFAS. In a similar vein, Lin et al. (2022) demonstrated a positive correlation between serum PFOS levels and cIMT in a Taiwanese cohort, with alterations in global DNA methylation (as indicated by modified 5-mdC/dG ratios) mediating this association [189,192]. Nonetheless, the application of a global methylation marker offers restricted mechanistic detail, and as an observational finding, this correlation is vulnerable to confounding factors. Notably, as Schillemans et al. (2023) have pointed out, while gestational and childhood PFAS-associated methylation changes are increasingly documented, significant gaps remain in our understanding of the mechanisms in adult populations [182].

Animal models have further enriched this paradigm. In Sprague–Dawley rats, Conley et al. (2023) employed a fixed-ratio PFAS mixture to reveal transcriptomic disruptions in maternal and fetal liver tissues, particularly in genes governing fatty acid metabolism and PPAR signaling. These transcriptomic changes were paralleled by subtle yet significant cardiac remodeling evidenced by increased left ventricular wall thickness, indicating that PFAS exposure may leave lasting epigenetic “bootprints” on cardiac gene networks [188,193]. More direct evidence comes from Min Qiu et al. (2024), who demonstrated that prenatal PFOS exposure in mouse models and human embryonic stem cell-derived cardiomyocytes leads to congenital heart defects, including septal anomalies and excessive ventricular trabeculation. These structural defects were associated with widespread hypermethylation in genomic regions critical for cardiac morphogenesis, implicating dysregulated activity of DNA methyltransferases (DNMTs) and TET dioxygenases [194,195].

These findings strongly supported the DOHaD framework, as early-life exposures to PFAS imprint the neonatal epigenome with lasting molecular alterations. Studies have shown that higher maternal PFAS concentrations are linked to congenital heart defects [195], while gestational exposure imprints both global methylation markers (e.g., LINE-1 elements) and gene-specific sites (e.g., ZFP57, NTN-1) associated with cardiac hypertrophy and development [43]. Genome-wide analyses, such as those conducted in a Faroese birth cohort, have revealed that prenatal PFOS exposure is associated with differential methylation at over 10,000 CpG sites in male neonates, highlighting pronounced sexual dimorphism in epigenetic responses [117]. Additional studies have linked early-life PFAS exposure with DNA methylation changes in genes regulating cardiovascular and renal function, thereby predisposing individuals to obesity, diabetes, and non-alcoholic fatty liver disease [196,197].

Recent epigenome-wide investigations in human tissues provide yet another dimension to this narrative. Everson et al. (2025) conducted an epigenome-wide study in placental tissue from 151 mother-infant dyads, identifying significant differential DNA methylation at 23 loci, many proximal to genes implicated in growth regulation and cardiometabolic processes (e.g., *EBF1* and *NAV2*) [17,121,198]. These analyses revealed a mixture of effects and notable sex-specific vulnerabilities, with female placentas displaying more pronounced epigenetic perturbations. Complementary work by Porfirio et al. (2024) in female rats showed that PFOS exposure resulted in declines in cardiac output and impaired vascular reactivity, concomitant with the downregulation of endothelial nitric oxide synthase (eNOS) at both the mRNA and protein levels, suggesting epigenetic mechanisms, possibly via promoter methylation or histone modifications, in the regulation of vascular function [199].

Reviews by Wen et al. (2023) have further delineated the broader epigenetic impact of PFAS, documenting concentration-dependent hypomethylation in response to PFOA and dysregulation of key epigenetic regulators, such as decreased TET1 alongside increased TET2 and TET3 expression [200]. In parallel, Feng et al. (2022) applied Bayesian kernel machine regression to link serum PFAS levels, particularly PFOS and PFNA, with cardiovascular outcomes such as myocardial infarction and stroke, positing that epigenetic modifications disrupting inflammation, lipid metabolism, and endothelial function may underpin these associations [201,202]. However, it is important to note that factors such as lifestyle choices or unmeasured co-exposures could potentially confound the observed relationships with cardiovascular outcomes.

Longitudinal studies have enriched our understanding of the temporal dynamics of PFAS-induced epigenetic reprogramming. Schmidt (2022) tracked genome-wide DNA methylation from cord blood at birth to peripheral blood at 12 years of age, identifying 435 differentially methylated CpG sites associated with gestational PFAS exposure, many of which persisted into adolescence, suggesting that early-life exposures leave enduring molecular “Post-it notes” predisposing individuals to cardiometabolic dysfunction [61,203]. However, such studies are limited by potential batch effects or population-specific factors. Importantly, in a hospital-based case–control study, Jiao et al. (2024) linked exposure to 19 PFAS congeners with an increased risk of congenital heart disease (CHD) in children, proposing that epigenetic mechanisms such as altered DNA methylation and chromatin architecture mediated by oxidative stress, endocrine disruption, and inflammation might underpin these associations [28,72,204]. It is essential to note that hospital-based case–control studies are associative in nature and may be affected by selection bias.

Integrating these diverse lines of evidence from cellular and animal studies to epidemiological and placental epigenomic investigations reveals a compelling paradigm in which PFAS exposure initiates a complex cascade of epigenetic modifications, including alterations in DNA methylation, histone modifications, and non-coding RNA expression. These molecular alterations disrupt vascular integrity, lipid metabolism, and insulin signaling, collectively heightening cardiometabolic disease risk [17,190,191,194,199,200,201,203,204,205,206]. Reviews by Ganakumar et al. (2024) and Zachariah et al. (2024) further emphasize that modifications in DNA methylation, histone marks, and microRNA profiles not only compromise metabolic and vascular function but may also facilitate the transgenerational transmission of disease risk [205,206].

#### 4.4.1. PFAS Exposure Epigenetics and the Pathogenesis of Preeclampsia

Preeclampsia is a severe hypertensive disorder of pregnancy that typically manifests after the 20th week of gestation and is characterized by elevated blood pressure, proteinuria, and end-organ damage affecting systems such as the renal and hepatic [138,139]. Epidemiological studies have established a correlation between the development of preeclampsia and PFAS exposure [207,208]. For instance, a retrospective C8 health study among Mid-Ohio Valley residents suggested an increased self-reported prevalence of eclampsia among individuals exposed to PFOA and PFOS [207,208]. In a case–control-focused survey, Bommarito et al. (2021) analyzed early-pregnancy maternal plasma from 75 preeclamptic cases and 75 controls at Brigham and Women’s Hospital in Boston. Preeclampsia was diagnosed per ACOG guidelines, defined by new or worsening hypertension (≥140 mmHg systolic or ≥90 mmHg diastolic) and proteinuria (>300 mg/24 h or a protein/creatinine ratio >0.20) after the 20th week of gestation. Quantification of nine legacy PFAS (PFHpA, PFOA, PFNA, PFDA, PFUnDA, PFHxS, PFOS, MeFOSAA, and PFOSA) alongside angiogenic biomarkers revealed that elevated PFOS and PFDA levels were associated with an increased likelihood of late-onset preeclampsia, although associations with early-onset preeclampsia were imprecise [138]. Notably, the relatively small sample size and the scope of this case–control study, which is limited to a single hospital, restrict its statistical power and generalizability.

Mechanistic studies further illuminate the potential biological underpinnings of these epidemiological observations. Ebel et al. (2023) employed a large register-based cohort to examine clinical endpoints, including gestational hypertension, preeclampsia, and gestational diabetes mellitus in women exposed to high PFAS levels via contaminated drinking water. Although their study did not directly measure epigenetic markers, it was framed by prior work from Blake and Fenton (2020). Epigenetic molecular perturbations disrupt critical cellular processes that are essential for proper vascular remodeling and placental development, including trophoblast invasion, angiogenesis, and apoptosis regulation [136,209]. Notably, Ebel et al. (2023) reported a marginally protective odds ratio (OR = 0.80; CI 0.63–1.03) in high-exposure settings, raising the possibility of a threshold effect or even a non-monotonic dose-response relationship that could mask subclinical epigenetic changes [209].

Animal and in vitro studies provide further mechanistic insight into the epigenetic effects of PFAS. Qiu et al. (2024) demonstrated that PFOS exposure during critical developmental windows leads to significant reprogramming of DNA methylation patterns via the suppression of TET1 and TET3 enzymes, resulting in a global hypermethylation state that impairs the expression of genes crucial for cardiomyocyte differentiation. Although their primary focus was fetal cardiac dysplasia, the mechanistic framework of altered TET-mediated demethylation offers a plausible parallel for placental development, where similar epigenetic misregulation could disrupt angiogenic and trophoblastic gene networks relevant to preeclampsia [194]. As noted earlier, such extrapolation from cardiac models to placental pathology remains hypothetical. Complementing these findings, Xie et al. (2024) conducted a prospective cohort study correlating maternal plasma PFAS levels with genome-wide and candidate gene-specific DNA methylation changes in placental tissue. Using RRBS and bisulfite amplicon sequencing (BSAS), they reported that exposures to PFOA and PFNA were associated with significant hypomethylation in the promoter region of *PLXDC1*, a gene critical for angiogenesis, as well as altered methylation in other candidate genes, including *IRS4*, *CHST7*, and *FGF13*. The hypermethylation of *FGF13*, which mirrors findings of its downregulated expression in early-onset preeclamptic placentas [210,211], further substantiates the hypothesis that PFAS-induced epigenetic modifications may compromise placental vascular function [71].

In addition to epigenetic modifications, PFAS-induced oxidative stress appears to play a central role in the pathogenesis of preeclampsia. Zhao et al. (2022) demonstrated in animal models and in vitro studies that PFOS exposure induces mitochondrial damage via activation of p38, MAPK, and JNK signaling pathways, thereby impeding trophoblast migration, invasion, and vascularization [139]. Epidemiological data also reinforce these mechanistic insights; Rylander et al. (2019) found that women in the highest quartiles of maternal serum PFAS concentrations (including PFOA, PFNA, and PFHxS) had markedly increased odds (ORs ranging from 1.83 to 2.18) of developing severe preeclampsia compared to those in the lowest quartile. Similarly, Wikström et al. (2019) reported that a doubling of early-pregnancy PFOS and PFNA levels was associated with a 38–53% higher risk of preeclampsia. Collectively, these findings suggest that PFAS exposure might trigger epigenetic alterations such as aberrant DNA methylation patterns affecting genes that govern placental vascularization and inflammatory responses, which contribute to the endothelial dysfunction characteristic of preeclampsia [212,213,214,215]. The accumulating body of evidence highlights a compelling yet intricate interplay among PFAS exposure, epigenetic modulation, and the onset of preeclampsia. Nevertheless, the majority of studies are predicated on single-time-point observational cohorts characterized by limited PFAS speciation and highly exposed populations, leading to mechanistic conclusions derived from animal or in vitro models, which lack direct human measurements.

#### 4.4.2. Bridging Maternal Exposure to Chronic Disease Risk

Chronic diseases, most notably cancer and cardio-metabolic disorders, and pervasive exposures to PFAS constitute a mechanistic nexus, with PFAS actively modulating cellular metabolic pathways and the epigenome to drive the etiopathogenesis of these conditions. Mechanistic studies, such as Imir (2021) and Imir and colleagues (2021), have demonstrated that PFAS exposure, particularly when compounded by a high-fat diet, enhances glycolytic flux and elevates the production of key metabolic intermediates such as pyruvate and acetyl-CoA. The increased availability of acetyl-CoA, a critical substrate for histone acetyltransferases, facilitates heightened acetylation of histone tails, most notably H3K27 and H3K9, thus promoting an open chromatin state that favors the transcription of oncogenic pathways. Concurrently, PFAS-mediated activation of nuclear receptors, such as PPARα, further integrates metabolic reprogramming with epigenetic deregulation, establishing a direct mechanistic link to tumor progression [165,216]. However, these are small-scale mechanistic studies derived from controlled animal models with high-fat diet exposures.

Epidemiological analyses by Zhang (2024) complement these mechanistic insights by implicating chronic PFAS exposure in heightened cancer risk. The accumulation of PFAS in human tissues has been associated with cellular disturbances, including oxidative stress and endocrine disruption, which are known to induce both global and gene-specific epigenetic alterations, such as aberrant DNA methylation and histone modification patterns [217]. However, the authors acknowledged that their cross-sectional design precludes causal inference and that PFAS measurements at a single time point may not accurately reflect exposure levels from 10–20 years earlier. In vitro studies bolster this framework. For example, Durham et al. (2023) found that PFOS triggers chronic diseases linked to epigenetic reprogramming by changing DNA methylation patterns and disrupting the spatial arrangement of repressive heterochromatin markers such as H3K9me3 and H3K27me3 while also increasing the levels of histone demethylases [163]. Similarly, Pierozan and colleagues (2020) demonstrated that exposure of non-tumorigenic breast epithelial cells to PFOS and PFOA results in heritable epigenetic alterations characterized by increased global DNA methylation and reductions in activating histone marks (e.g., H3K9ac and H3K4me3), which disrupt cell-cycle regulation and promote malignant transformation [81]. The dualistic nature of PFAS-induced epigenetic modifications, encompassing both hypo- and hypermethylation events that can activate oncogenic pathways or suppress tumor suppressor genes [218], is further underscored by parallels drawn with mechanisms implicated in cancer immunotherapy resistance [219].

### 4.5. PFAS-Mediated Epigenetic Modifications and Immune Dysfunction

PFAS disrupts the immune balance through complex epigenetic changes, heightening the risk of infections and other immune diseases. Current research identifies essential epigenetic processes that collectively reorganize immune gene networks. For instance, Ahmad and colleagues (2021) [220] demonstrated that PFOA accumulates in the lungs, and exposure leads to significant hypomethylation of CpG islands in the promoter region of Tmprss2. Furthermore, DNMTs and TET enzymes were also downregulated, which is paralleled by Bulka et al.’s (2022) finding of hypomethylation in key immune-related genes such as *ACE2* and *TMPRSS2*, potentially heightening viral susceptibility [221]. These DNA methylation changes may interact with alterations in histone modification patterns, as documented in animal models and in vitro studies [163,220], which modulate chromatin accessibility at loci and are crucial for immune regulation. Such crosstalk between methylation and histone marks suggests an integrated regulatory axis that could be further elucidated using network analysis approaches. Ahmad et al. (2021) [220] administered supra-physiological doses of PFOA (5–20 mg/kg/day) to CD1 mice, using bulk-tissue methylation assays that lack cell-type specificity [220]. Hence, caution must be exercised when extrapolating findings to human pulmonary epigenetics, particularly considering the species-specific pharmacokinetics of PFOA and the distinctions between experimental and environmental exposures. Likewise, Bulka et al. (2022) [221] compiled diverse cellular and observational data regarding ACE2/TMPRSS2 hypomethylation, which provides valuable context, yet underscores the necessity of utilizing controlled in vivo infection models to validate the functional impacts on viral susceptibility.

Beyond these modifications, non-coding RNAs also serve as key regulators of immune function under PFAS exposure. Altered expression profiles of microRNAs and long non-coding RNAs, as reported by Li and colleagues (2022) [87] and Xu and colleagues (2020) [93], have been implicated in reprogramming inflammatory signaling pathways and directing immune cell differentiation [87,93,163]. This triad of epigenetic changes contributes to the dysregulation of both T- and B-cell compartments, with studies by Liang and co-workers (2022) and Rudzanová and co-workers (2023) reporting diminished immunoglobulin production and shifts in lymphocyte subset distributions [222,223]. Notably, the downregulation of transcription factors critical for plasma cell maturation, such as EBF1 and PAX5, further compounds the reduction in antibody titers and may underlie decreased vaccine responsiveness [221,223]. Nonetheless, these studies (Li et al., 2023 [87], and Xu, 2020 [93]) are limited by small sample sizes and a dependence on single-time-point analyses within mixed immune cell populations, lacking in vivo functional validation of the candidate miRNAs and lncRNAs.

PFAS-mediated epigenetic alterations also extend to the regulation of cytokine networks and inflammasome activation. For example, Liang and colleagues (2022) [222] observed an upregulation of IL-1β and TNF-α via NF-κB and AIM2 pathways, while Papadopoulou and colleagues (2021) [166] documented a seemingly paradoxical profile characterized by simultaneous increases in IL-1β with suppressed levels of IFN-α and IL-8. These contrasting cytokine responses underscore the complexity of PFAS-induced immune modulation and highlight the need for detailed dose-response and temporal analyses. Notably, emerging data on telomere dynamics, including PFAS-associated telomere shortening [222] and alterations in epigenetic aging metrics (Niemiec et al., 2023), suggest that these exposures may accelerate immune senescence, even as some compensatory mechanisms, such as the deceleration of epigenetic aging in neonatal cord blood, are observed [224]. However, these models depend on in vitro and in vivo hepatocellular carcinoma (HCC) proliferation assays as well as promoter-bias studies conducted in aquatic species, thereby emphasizing the necessity for mechanistic validation based on human tissue.

Comparative analyses across study designs further emphasize the heterogeneous nature of PFAS effects. While controlled experimental models provide robust mechanistic insights, human epidemiological studies often reveal variable outcomes. For instance, despite several reports linking PFAS exposure to reduced vaccine efficacy and an elevated risk of severe infections [163,220], cohorts such as those examined by Sevelsted and colleagues (2023) have shown minimal associations between PFAS levels and early childhood infections [225]. These discrepancies may be attributable to factors including exposure windows, genetic variability, nutritional status (e.g., folate availability), and the intrinsic differences among PFAS congeners, each of which can differentially modulate nuclear receptor pathways [226].

Notably, PFAS-induced epigenetic reprogramming of immune-related genes can heighten susceptibility to infections and inflammatory disorders, potentially exacerbating autoimmune conditions over time. Emerging data also point to an increased risk of carcinogenesis via epigenetically driven pathways, including the silencing of tumor suppressor genes and the activation of oncogenic networks [227]. Current literature presents a nuanced yet largely convergent narrative: PFAS exposure induces a complex network of epigenetic modifications that collectively disrupt immune homeostasis. The interplay between DNA methylation, histone modifications, and non-coding RNA regulation not only reprograms immune gene expression but also predisposes individuals to infections and autoimmune conditions.

## 5. Future Directions

Although robust associations between PFAS exposure and changes in DNA methylation, histone modifications, and non-coding RNA expression have been documented, further mechanistic studies employing advanced experimental models such as CRISPR/dCas9-based epigenetic editing, refined epidemiological designs, and integrated hormone profiling are essential to establish causative pathways and to guide personalized intervention strategies [102,105,106]. Future research on PFAS-induced epigenetic alterations must embrace an integrative, multi-disciplinary approach to resolve the intricate molecular pathways linking early-life exposures to long-term health outcomes. Longitudinal studies that combine comprehensive exposure assessments with cutting-edge multi-omics methodologies such as epigenome-wide association studies, transcriptomics, and metabolomics are essential for disentangling the complex interplay of genetic predisposition, environmental toxicants, and epigenetic remodeling [148,157]. Among these, single-cell multi-omics platforms stand out as particularly transformative. By simultaneously capturing transcriptomic, epigenomic, and even proteomic profiles at the individual cell level, these methods (e.g., scRNA-seq coupled with scATAC-seq and single-cell CUT&Tag) provide an unprecedented resolution of cell-specific responses and heterogeneity. Such efforts should aim to delineate compound-specific epigenetic signatures, explore the reversibility of these modifications, and reconcile epidemiological inconsistencies, particularly those pertaining to sexual dimorphism and age-dependent susceptibility [134,152].

Simultaneously, the development of experimental models that accurately mimic realistic exposure scenarios will be vital. Advanced in vitro systems, coupled with in vivo models that incorporate advanced genome editing and epigenetic editing, can provide mechanistic insights into the disruption of DNA methylation, histone modification dynamics, and non-coding RNA networks. These models could further clarify how PFAS-mediated perturbations in one-carbon metabolism and endocrine signaling contribute to persistent alterations in gene expression, thereby driving disease pathogenesis from neurodevelopmental disorders to metabolic and immune dysfunctions [150,228].

The translational potential of these findings should not be overlooked. Given the persistence of PFAS in the environment and their capacity to induce long-lasting epigenetic reprogramming, it is imperative to investigate interventional strategies that might mitigate these effects. Nutritional supplementation (e.g., folate) and pharmacological modulation of epigenetic enzymes represent promising avenues for intervention. Future studies should rigorously evaluate whether such strategies can restore normal epigenetic patterns and, by extension, improve long-term health outcomes in exposed populations.

To facilitate these endeavors, the establishment of standardized methodologies and innovative analytical frameworks is crucial. Potential computational tools for future studies include artificial intelligence that models the network of epigenetic modifications induced by different PFAS compounds, as well as summarizing the key mechanistic gaps and proposed research directions. Such visual aids would enhance clarity and foster cross-disciplinary communication among toxicologists, molecular biologists, and public health experts. Ultimately, a concerted effort integrating mechanistic, epidemiological, and translational research will be essential to not only elucidate the underlying biology of PFAS-induced epigenetic disruptions but also to inform robust public health policies aimed at mitigating their long-term adverse effects.

## 6. Conclusions

The growing body of evidence underscores the significant impact of in utero PFAS exposure on epigenetic regulation, with profound implications for fetal development and long-term health outcomes. Through mechanisms involving DNA methylation, histone modifications, and non-coding RNA dysregulation, PFAS exposure has been shown to alter critical gene regulatory networks, leading to disruptions in metabolic homeostasis, neurodevelopment, immune function, and endocrine signaling. Studies have consistently demonstrated that PFAS compounds can induce changes in DNA methylation at imprinted loci such as IGF2 and MEST, thereby influencing fetal growth trajectories and increasing susceptibility to metabolic disorders. Furthermore, histone-modifying enzymes such as HATs and HDACs appear to be particularly vulnerable to PFAS-induced dysregulation, resulting in widespread chromatin remodeling events that impact gene expression. Additionally, alterations in miRNA and lncRNA profiles further illustrate the intricate ways in which PFAS disrupt gene expression regulation at multiple molecular levels.

Critical knowledge gaps remain despite significant advancements in our understanding of PFAS-induced epigenetic modifications. The persistence of these alterations across the lifespan, their potential to be inherited transgenerationally, and sex-specific susceptibilities to PFAS exposure warrant further investigation. Additionally, the differential effects of various PFAS congeners on epigenetic programming suggest that compound-specific regulatory mechanisms may exist, necessitating further research to elucidate their distinct molecular pathways. Standardization in methodological approaches, including high-resolution epigenome-wide association studies (EWAS) and integrative multi-omics analyses, will be essential to bridge these gaps and establish causative links between PFAS exposure and disease risk.

From a public health and regulatory standpoint, these findings highlight the urgent need for policies aimed at reducing PFAS exposure, particularly among vulnerable populations such as pregnant individuals and developing fetuses. As PFAS contamination remains pervasive in the environment and human biological systems, intervention strategies must extend beyond exposure reduction to include potential therapeutic avenues that target epigenetic modifications. Future research should explore the feasibility of epigenetic interventions, such as dietary supplementation with methyl donors, pharmaceutical modulation of chromatin modifiers, and the use of CRISPR-based epigenetic editing technologies to mitigate the adverse effects of PFAS-induced epigenetic disruptions.

## Figures and Tables

**Table 1:** Summary of human cohort studies on prenatal PFAS exposure and alterations in DNA methylation.

Author(s), Year. [Reference]	DNA Methylation Measurement	Primary Findings	Study Design	Population Characteristics	Exposure
Villanger et al., 2023 [67]	- Biological sample used: Blood from pregnant women and cord blood from newborn children - Specific methylation types measured: 5-methylcytosine (5-mC) and 5-hydroxymethylcytosine (5-hmC) - Adjusted elastic net regression and quantile g-computation approach were used for analysis	- Specific PFAS compounds showing significant associations: PFHxS (important for 5-mC in both mothers and infants), PFOS (important for 5-hmC in mothers) - No joint effect of PFAS mixtures on DNA methylation markers - Subgroup analyses: Relationship varied with seafood intake and PFDA concentrations for mothers; maternal education level, seafood intake, and smoking during pregnancy for infants - No specific numbers of statistically significant methylation sites, direction of changes, statistical significance levels, or effect sizes/regression coefficients provided	Prospective cohort study; Mother-infant cohort study (part of the Norwegian Mother, Father, and Child Cohort Study)	- Total number of participants: 634 pregnant women - Number of mother-infant pairs: 634 - Geographical location of the study: Norway Recruitment period: 2010–2014 - Maternal characteristics: Gestation week ~18	- Specific PFAS compounds measured: PFHxS, PFOS (seven PFAS measured, but only these two specified) - Biological sample used for PFAS measurement: maternal blood, cord blood - Timing of exposure measurement: gestation week ~ 18
Robinson et al., 2021 [68]	- Biological sample used: DNA extracted from dried blood spots (DBS) - Measurement technique: Infinium MethylationEPIC BeadChip - Specific genomic regions or genes analyzed: Individual CpG sites, including cg15557840 near SCRT2, SRXN1; cg19039925 in GVIN1 in boys; cg05754408 in ZNF26 in girls; cg03278866 within PTBP1 - Methylation quantification method: Robust linear regression examining associations with DNA methylation at individual CpG sites - Unique aspect: Analysis included 2242 CpG sites identified as Correlated Regions of Systemic Interindividual Variation (CoRSIVs)	- Number of statistically significant methylation sites: 4 (cg15557840, cg19039925, cg05754408, cg03278866) - Specific PFAS compounds showing significant associations: PFOA and PFOS - Statistical significance levels: FDR < 0.05 - Sex-specific analyses: PFOS associated with cg19039925 in boys and cg05754408 in girls - Notable non-significant trends: Limited evidence of association overall	Cohort study; Mother-infant cohort study	- Recruitment period: 2008 and 2010 - Total number of participants: 597 neonates - Infant characteristics: Sex and plurality mentioned as covariates	- Specific PFAS compounds measured: PFOA, PFOS - Biological sample used for PFAS measurement: Newborn dried blood spots (DBS) - Measurement method/technique: High-performance liquid chromatography/tandem mass spectrometry (LC-MS/MS) - Timing of exposure measurement: At birth (implied from newborn DBS) - Concentration ranges or summary statistics: >90th percentile concentrations were analyzed
Miura et al., 2018 [69]	- Biological sample used: Cord blood - Specific methylation types measured: 5-methylcytosine - Measurement technique: Illumina HumanMethylation 450 BeadChip - Specific genomic regions or genes analyzed: 485,577 CpGs across the genome - Methylation quantification method: Beta-values calculated from signal intensities - Unique aspects: Identification of differentially methylated regions (DMRs) using bumphunter function	- Number of statistically significant methylation sites: Four DMPs with FDR < 0.05 - Specific PFAS compounds showing significant associations: PFOS and PFOA - Direction of methylation changes: Up-methylation for PFOS, down-methylation for PFOA - Statistical significance levels: FDR < 0.05 for DMPs, FWER < 0.1 for DMRs	Prospective cohort study; Mother-child cohort study	Number of mother-infant pairs: 190 - Geographical location of the study: Sapporo, Japan - Recruitment period: 2002–2005 Inclusion/exclusion criteria: Inclusion—pregnant women at 23–35 weeks of gestation; Exclusion—miscarriage, stillbirth, relocation, voluntary withdrawal, multiple births - Maternal characteristics: Average age 29.7 ± 4.8 years - Infant characteristics: Sex distribution—44.2% male	- Specific PFAS compounds measured: PFOS, PFOA - Biological sample used for PFAS measurement: Maternal serum - Measurement method/technique: Column-switching liquid chromatography-tandem mass spectrometry (LC-MS/MS) - Timing of exposure measurement: Between 24 and 41 weeks of gestational age - Concentration ranges or summary statistics: Median PFOS: 5.2 ng/mL (3.8 to 7.1), Median PFOA: 1.4 ng/mL (0.9 to 2.1)
Liu et al., 2021 [70]	- Biological sample used: Cord blood at delivery and peripheral leukocyte DNA at age 12 years - Measurement technique: Illumina HumanMethylation EPIC BeadChip - Specific genomic regions or genes analyzed: Loci mapped to genes such as AGAP1, HPSE2, HABP2, RNF13, RADIL, and TMEM56 - Methylation quantification method: Associations analyzed using generalized estimating equations	- Number of statistically significant methylation sites: 35 - Specific PFAS compounds showing significant associations: PFOS (5 loci), PFOA (10 loci), PFHxS (7 loci), PFNA (13 loci) - Statistical significance levels: q-value 0.05 for overall loci, q-value 0.01 for specific loci	Prospective mother-child cohort study	- Total number of participants: 532 (266 mothers and 266 children) - Number of mother-infant pairs: 266 - Geographical location of the study: Cincinnati, OH - Recruitment period: 2003–2006) - Maternal characteristics: Gestational age at serum measurement ~ 16 weeks	- Specific PFAS compounds measured: PFOA, PFOS, PFNA, PFHxS - Biological sample used for PFAS measurement: Maternal serum - Timing of exposure measurement: ~16 weeks gestation
Everson et al., 2025 [17]	- Biological sample used: Human placental tissues - Specific methylation types measured: Implied 5-methylcytosine (via bisulfite conversion) - Measurement technique: Illumina MethylationEPIC Beadarray - Specific genomic regions or genes analyzed: Epigenome-wide (broad analysis across the genome) - Methylation quantification method: Functional normalization and beta-mixture quantile (BMIQ) normalization	- Number of statistically significant methylation sites: 23 loci - Specific PFAS compounds showing significant associations: PFHxS (11 loci), PFNA (5 loci), PFOS (4 loci), PFOA (2 loci), PFDA (1 locus) - Direction of methylation changes: Both increased and decreased (6 loci increased, 6 loci decreased) - Statistical significance levels: FDR *q*-values < 0.05 - Sex-specific analyses: More methylation perturbations in females than males, particularly for PFHxS and PFOS	Prospective longitudinal observational cohort study; Mother-infant cohort study	- Total number of participants: 151 - Number of mother-infant pairs: 151 - Geographical location of the study: Little Rock, Arkansas - Recruitment period: 2010 to 2014 - Inclusion/exclusion criteria: Inclusion—mothers at least 21 years old, second parity; Exclusion—pre-existing medical conditions, sexually transmitted infections, medical complications, smoking or alcohol use during pregnancy, medication use known to influence fetal growth, conceptions aided with fertility treatment - Maternal characteristics: Mean age 30.5 years (SD = 3.42), most had a college degree, most self-identified as White - Infant characteristics: 63 females and 88 males, average gestation of 39.3 weeks (range 36.4–41.4 weeks)	- Specific PFAS compounds measured: PFHxA, PFHxS, PFHpA, PFOA, PFOS, PFOSA, MePFOSAA, PFNA, PFDA, PFDS, PFUnDA, PFDoDA, PFPeA, EtPFOSAA, HFPO-DA (Gen X), PFHpS, PFBS - Biological sample used for PFAS measurement: Human placental tissue - Measurement method/technique: High-performance liquid chromatography-tandem mass spectrometry (LC-MS/MS) with electrospray ionization - Timing of exposure measurement: Recruitment of pregnant women prior to gestational week 10 (2010–2014) - Concentration ranges or summary statistics: Five PFAS (PFHxS, PFOS, PFOA, PFNA, and PFDA) were detectable in over 70% of placental samples; PFOS had the highest average concentrations
Xie et al., 2024 [71]	- Biological sample used: Placental tissue - Specific methylation types measured: Likely 5-methylcytosine (inferred from bisulfite sequencing) - Measurement technique: Reduced representation bisulfite sequencing for genome-wide analysis; bisulfite amplicon sequencing for targeted gene analysis - Specific genomic regions or genes analyzed: CHST7, FGF13, IRS4, PHOX2A, PLXDC1 - Methylation quantification method: Sequencing	- Specific PFAS compounds showing significant associations: PFOA, PFNA, PFTrDA, PFDoA - Direction of methylation changes: - PFOA associated with hypomethylation of IRS4 and PLXDC1 - PFNA associated with hypomethylation of PLXDC1 - Positive associations (increased methylation) of CHST7 with PFTrDA and IRS4 with PFDoA and PFTrDA	Prospective cohort study; Mother-infant cohort study	- Total number of participants: implied 690 from 345 mother-infant pairs) - Recruitment period: April–December 2012 - Number of mother-infant pairs: 345 - Maternal characteristics: (PFAS measured during early pregnancy) - Infant characteristics: (development assessed at six months)	- Specific PFAS compounds measured: PFOA, PFNA, PFTrDA, PFDoA - Biological sample used for PFAS measurement: maternal plasma - Timing of exposure measurement: Early pregnancy
Liu et al., 2021 [61]	- Biological sample used: Cord blood and peripheral leukocytes at 12 years of age - Specific methylation types measured: likely 5-methylcytosine at CpG sites - Measurement technique: Illumina HumanMethylation EPIC BeadChip - Specific genomic regions or genes analyzed: CpG sites associated with cancers, cognitive health, cardiovascular disease, and kidney function - Methylation quantification method: Associations analyzed using linear regression with generalized estimating equations	- Number of statistically significant methylation sites: 435 CpG sites - Specific PFAS compounds showing significant associations: PFOS (2 CpGs), PFOA (12 CpGs), PFHxS (8 CpGs), PFNA (413 CpGs) - Statistical significance levels: *q* < 0.05 - Subgroup analyses: Little evidence of age-specific differences	Prospective longitudinal mother-child cohort study	- Geographical location of the study: Cincinnati, Ohio - Recruitment period: 2003–2006	- Specific PFAS compounds measured: PFOA, PFOS, PFNA, PFHxS - Biological sample used for PFAS measurement: Maternal serum - Timing of exposure measurement: During pregnancy
Petroff et al., 2023 [20]	- Biological sample: Cord blood (nucleated cells such as leukocytes and nucleated red blood cells) - Specific methylation types measured: 5-methylcytosine (5-mC) and 5-hydroxymethylcytosine (5-hmC) - Measurement technique: Illumina MethylationEPIC BeadChip - Specific genomic regions or genes analyzed: Over 850,000 CpG sites - Methylation quantification method: MLML method for estimating 5-mC, 5-hmC, and unmethylated cytosines - Unique aspects: Use of oxidative bisulfite conversion to specifically measure 5-hmC	- Number of statistically significant methylation sites: - Total methylation: PFHxS 12 sites; PFOS 19 sites; PFOA 2 sites; PFNA 3 sites; PFDA 4 sites. - 5-mC and 5-hmC: Thousands of sites for PFHxS, PFOS, PFNA, PFDA, PFUnDA, and MeFOSAA. - Specific PFAS compounds showing significant associations: PFHxS, PFOS, PFOA, PFNA, PFDA, PFUnDA, MeFOSAA. - Direction of methylation changes: Decreased 5-hmC and increased 5-mC. - Statistical significance levels: q < 0.05. - Sex-specific analyses: Significant sex interactions for all PFAS; specific numbers of significant sites in males and females	Prospective birth cohort study; Mother-infant cohort study	- Total number of participants: 309 - Number of mother-infant pairs: 288 - Geographical location of the study: University of Michigan Von Voigtlander Women’s Hospital - Recruitment period: 2010 to 2019 - Inclusion/exclusion criteria: Inclusion—at least 18 years old, singleton pregnancy, between 8 and 14 weeks gestation, intended delivery at the University of Michigan Hospital - Maternal characteristics: Average age: 31.8 years, Mean baseline weight: 69–70 kg, Average baseline BMI: 25.5–25.8 - Infant characteristics: Sex distribution—Female: 72, Male: 69	- Specific PFAS compounds measured: MeFOSAA, PFOSA, PFHxS, PFHpA, PFOA, PFOS, PFDA, PFNA, PFUnDA - Biological sample used for PFAS measurement: Maternal plasma - Measurement method/technique: On-line solid phase extraction coupled with high-performance liquid chromatography-isotope dilution tandem mass spectrometry (LC-MS/MS) - Timing of exposure measurement: First trimester - Concentration ranges or summary statistics: Geometric mean concentrations were 3.2 µg/L for PFHxS, 5.3 µg/L for PFOS, 1.1 µg/L for PFOA, 0.37 µg/L for PFNA, and 0.12 µg/L for PFDA
Starling et al., 2020 [72]	- Biological sample: Umbilical cord blood - Measurement technique: Illumina HumanMethylation450 array - Specific genomic regions or genes analyzed: DMPs and DMRs, including genes such as *TJAP1*, *RPTOR*, *PON1*, *PON3*, *CIDEB*, *NR1H2*, *RASL11B*, *RNF39* - Methylation quantification method: Evaluation of DMPs at FDR < 0.05 and identification of DMRs using comb-p with Šidák-adjusted *p* < 0.05	- Number of statistically significant methylation sites: 1 DMP (cg18587484) - Specific PFAS compounds showing significant associations: PFOA is mentioned - Statistical significance levels: FDR < 0.05 for DMP; Šidák-adjusted *p* < 0.05 for DMRs	Prospective cohort study; Mother-infant cohort study	- Total number of participants: 583 mother-infant pairs - Number of mother-infant pairs: 583 - Geographical location of the study: (suggested U.S. based on context) - Recruitment period: 2009–2014 - Maternal characteristics: PFAS measured at median 27 weeks of gestation	- Specific PFAS compounds measured: PFOA is mentioned - Biological sample used for PFAS measurement: Maternal serum - Timing of exposure measurement: Median 27 weeks of gestation - Concentration ranges or summary statistics: Below the median for females in the U.S. general population
Kobayashi et al., 2017 [73]	- Biological sample used: Cord blood DNA - Specific methylation types measured: likely 5-methylcytosine due to bisulfite sequencing - Measurement technique: Bisulfite pyrosequencing - Specific genomic regions or genes analyzed: Two differentially methylated regions (DMRs) within IGF2/H19 locus, as well as LINE1 - Methylation quantification method: Bisulfite pyrosequencing	- Number of statistically significant methylation sites: 1 (IGF2) - Specific PFAS compounds showing significant associations: PFOA - Direction of methylation changes: Decreased - Statistical significance levels: ß = −1.61, 95% CI: −3.00 to −0.22 (for PFOA and IGF2) - Effect sizes or regression coefficients: ß = −1.61 for IGF2 with PFOA - No sex-specific or subgroup analyses were mentioned	Prospective mother-child cohort study	- Total number of participants: 514 pregnant women - Number of mother-infant pairs: 235 - Geographical location of the study: Sapporo, Japan - Recruitment period: 2002–2005	- Specific PFAS compounds measured: PFOA, PFOS - Biological sample used for PFAS measurement: maternal serum - Measurement method/technique: LC–MS/MS - Concentration ranges or summary statistics: Median concentrations of PFOS: 5.0 ng/mL, PFOA: 1.4 ng/mL

## Data Availability

No new data were created or analyzed in this study. Data sharing does not apply to this article.

## Abbreviations

The following abbreviations are used in this manuscript:

PFAS	Per- and polyfluoroalkyl substances
PFBS	Perfluorobutane sulfonate
PFHpA	Perfluoroheptanoic acid
PFHpS	Perfluoroheptane sulfonic acid
PFHxA	Perfluorohexanoic acid
PFHxS	Perfluorohexane sulfonic acid
PFNA	Perfluorononanoic acid
PFOA	Perfluorooctanoic acid
PFOSA	Perfluorooctane sulfonamide
PFOS	Perfluorooctane sulfonic acid
PFPeA	Perfluoropentanoic acid
PFDS	Perfluorodecane sulfonic acid
PFDoDA	Perfluorododecanoic acid
PFTrDA	Perfluorotridecanoic acid
PFUnDA	Perfluoroundecanoic acid
MeFOSAA	Methyl-perfluorooctane sulfonamido acetic acid
EtPFOSAA	Ethyl-perfluorooctane sulfonamido acetic acid
HFPO-DA	Hexafluoropropylene oxide dimer acid
HFPO-TA	Hexafluoropropylene oxide trimer acid
HATs	Histone acetyltransferases
HDACs	Histone deacetylases
IGF2	Insulin-like growth factor 2
MEST	Mesoderm-specific transcript
lncRNAs	Long non-coding RNAs
DNMTs	DNA methyltransferases
DMRs	Differentially methylated regions
PPAR-α	Peroxisome proliferator-activated receptor—alpha
MEG3	Maternally expressed gene 3
PTX3	Pentraxin 3
ROS	Reactive oxygen species
BDNF	Brain-derived neurotrophic factor
CREB	cAMP-responsive element binding protein
ER	Estrogen receptor
DOHaD	Developmental Origins of Health and Disease
HFPO-TA	Hexafluoropropylene oxide trimer acid
WGBS	Whole-genome bisulfite sequencing
UPLC–MS/MS	Ultra-performance liquid chromatography–tandem mass spectrometry
EWAS	High-resolution epigenome-wide association studies
CHD	Congenital heart disease
RRBS	Reduced representation bisulfite sequencing
ChIP-seq	Chromatin immunoprecipitation sequencing
SAM	S-adenosylmethionine
SUMO	Small ubiquitin-related modifier
